# The Advantages of Flexibility: The Role of Entropy in Crystal Structures Containing C–H···F Interactions

**DOI:** 10.1021/acs.cgd.4c00042

**Published:** 2024-02-19

**Authors:** Cameron J. G. Wilson, Jan Plesniar, Heike Kuhn, Jeff Armstrong, Peter A. Wood, Simon Parsons

**Affiliations:** † Centre for Science at Extreme Conditions, School of Chemistry, The University of Edinburgh, King’s Buildings, West Mains Road, Edinburgh EH9 3FJ, U.K.; ‡ ISIS Facility, STFC, Rutherford Appleton Laboratory, Chilton, Didcot, Oxfordshire OX11 0QX, U.K.; § The Cambridge Crystallographic Data Centre, 12 Union Road, Cambridge CB2 1EZ, U.K.

## Abstract

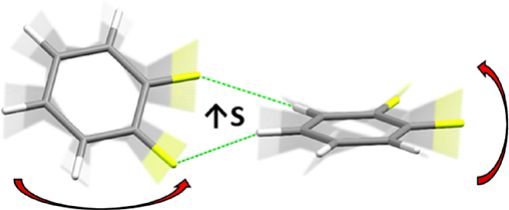

Molecular crystal structures are often interpreted in terms of strong, structure directing, intermolecular interactions, especially those with distinct geometric signatures such as H-bonds or π-stacking interactions. Other interactions can be overlooked, perhaps because they are weak or lack a characteristic geometry. We show that although the cumulative effect of weak interactions is significant, their deformability also leads to occupation of low energy vibrational energy levels, which provides an additional stabilizing entropic contribution. The entropies of five fluorobenzene derivatives have been calculated by periodic DFT calculations to assess the entropic influence of C–H···F interactions in stabilizing their crystal structures. Calculations reproduce inelastic neutron scattering data and experimental entropies from heat capacity measurements. C–H···F contacts are shown to have force constants which are around half of those of more familiar interactions such as hydrogen bonds, halogen bonds, and C–H···π interactions. This feature, in combination with the relatively high mass of F, means that the lowest energy vibrations in crystalline fluorobenzenes are dominated by C–H···F contributions. C–H···F contacts occur much more frequently than would be expected from their enthalpic contributions alone, but at 150 K, the stabilizing contribution of entropy provides, at −10 to −15 kJ mol^–1^, a similar level of stabilization to the N–H···N hydrogen bond in ammonia and O–H···O hydrogen bond in water.

## Abstract 2

The stabilization provided by entropy in weakly bonded crystal structures can be similar to that provided by H-bonding.

## 1 Introduction

The structures formed by materials under different conditions are the consequence of minimization of free energy, which can be interpreted as the result of a competition between enthalpic and entropic contributions. For example, sublimation, a change in phase from solid to gas, occurs, to a first approximation, when the temperature is high enough for the entropy of the gaseous state to overcome the enthalpic contribution made by the intermolecular interactions in the solid. Likewise, the crystal structures adopted by a compound are the result of a competition between the enthalpic and entropic characteristics of the intermolecular interactions.

The directing influence of strong intermolecular interactions such as hydrogen bonds in the determination of stable packing configurations in crystal structures can often be readily identified. Motifs, such as the familiar dimers formed between carboxylic acid groups, occur so frequently and consistently that they are considered to be supramolecular synthons that can be used in strategies to engineer crystal structures with predetermined architectures.^1^ Such interactions typically have energies in the range of tens of kJ mol^–1^.^2^ The role of weak interactions is more subtle and open to different interpretations. Among these are weak C–H···halogen interactions, sometimes referred to as a class of weak hydrogen bond,^3^ which have molecule–molecule energies well below 10 kJ mol^–1^.

A study by Taylor has investigated the importance of weak C–H···X (X = O, N, F, Cl) interactions in crystal packing.^4^ The frequency of formation of specific interactions was evaluated in the context of molecular surface area analysis to assess the extent to which interactions are formed because of their favorable characteristics as opposed to being formed randomly. A metric *R*_F_ for primary (shortest) interactions was defined and used to measure the favorability of different classes of interaction, with values greater than unity indicating greater than random occurrence.

Charge density analysis has shown that there is an intrinsic polarization of the electron density on the fluorine atoms,^5^ which promotes its participation in C–H···F, C–F···F–C, and C–F···π interactions in the solid state,^6^ though overall, these interactions have weak, largely dispersion based, rather than electrostatic, enthalpic contributions. Nevertheless, the value of *R*_F_ for C–H···F intermolecular interactions in Taylor’s study was, at 3.5, strikingly higher than either C–H···O (2.7) or C–H···N (3.0) interactions, which occasionally appear in lists of supramolecular synthons.^3^ Our aim in this paper is to offer an explanation of why C–H···F interactions should occur so frequently.

It has been argued^7−9^ that although individual C–H···F interactions are energetically weak, the presence of many of them becomes significant through a cooperative effect. Although these interaction energies are clearly significant, the free energy of a crystal structure is also influenced by other contributions, which can exert a decisive influence on phase stability. For example, volume minimization is the most important driving force of high-pressure phase transitions, in which the change in lattice energy is often found to be positive.^10^ A computational study on the role of entropy by Nyman and Day on 1061 experimentally determined structures of 508 polymorphic organic molecules has shown that vibrational contributions dominated by entropy differences contribute to significant differences in free energy. These were shown to be large enough in 9% of their polymorph pairs to cause a reranking of polymorph stability.^11^ Kieslich et al. have also demonstrated the importance of entropy in hybrid organic–inorganic perovskites as a balance and interplay with hydrogen bonding interactions.^12^ The ability of entropy to support negative volume changes in the presence of positive lattice energy changes has also been noted in a high-pressure polymorph of salicylamide.^13^

**Figure 1:**
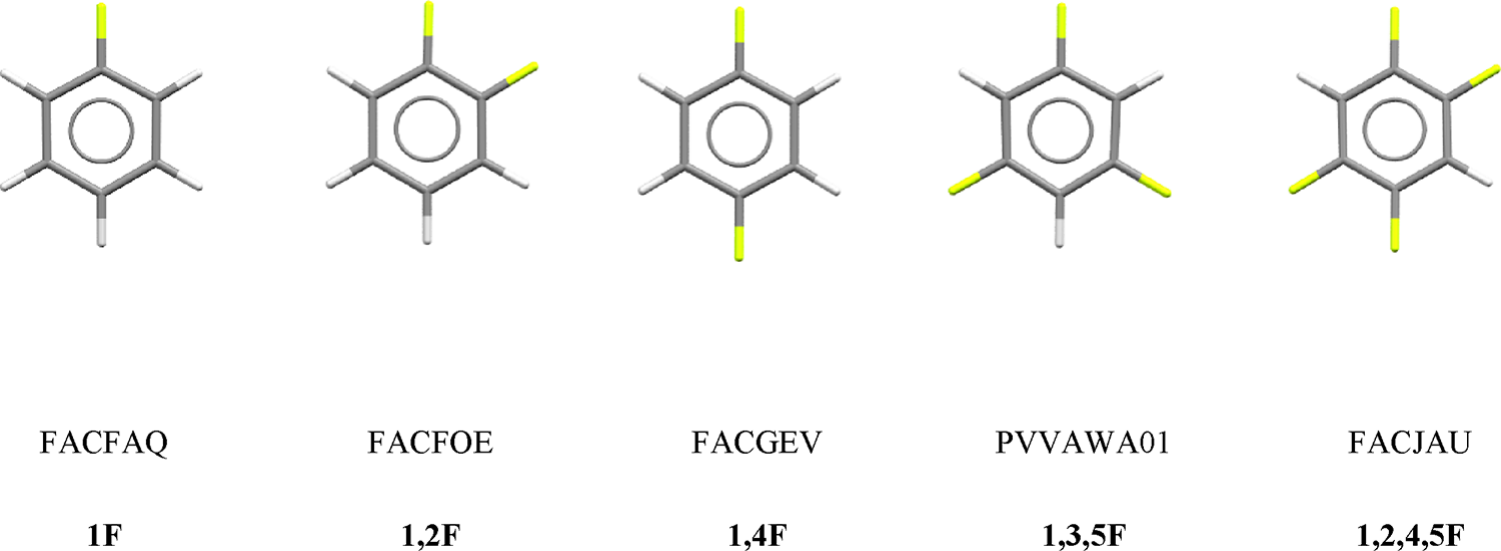
Fluorobenzene derivatives investigated in this study and the definitions of the compound codes used in the text.

In this paper, we suggest on the basis of calculations on a series of fluorobenzene derivatives (Figure 1) that the shape and energetic characteristics of the potentials of C–H···F interactions leads to high deformability and low vibrational frequencies, so that crystal structures in which they occur are favored not only by the enthalpic influence of their interaction energies but also by entropy.

## 2 Materials and Methods

### 2.1 Structures Studied

Calculations were carried out on five fluorobenzene derivatives: monofluorobenzene (Cambridge Structural Database^14^ refcode FACFAQ, **1F** hereafter), 1,2-difluorobenzene (FACFOE, **1,2F**), 1,4-difluorobenzene (FACGEV, **1,4F**), 1,3,5-trifluorobenzene (PVVAWA01, **1,3,5F**), and 1,2,4,5-tetrafluorobenzene (FACJAU, **1,2,4,5F**). These structures, which were all published in a landmark study by Thalladi et al., were used to define the initial starting geometries for the DFT optimizations described below.^9^ The five structures selected from Thalladi et al.’s study sample different classes of interaction geometry while minimizing computational expense by choosing in all cases except **1,2F**, molecules that occupy special positions, enabling symmetry to be exploited in the calculations.

### 2.2 DFT Geometry Optimization

The crystal structures of the compounds shown in Figure 1 were geometry-optimized using periodic density functional theory (DFT) with CASTEP through the Materials Studio interface.^15,16^ The Perdew–Burke–Ernzerhof (PBE)^17^ exchange-correlation functional and norm-conserving pseudopotentials^15^ were used with a basis set cutoff energy of 990 eV and a **k**-point spacing of 0.04 Å^–1^, which converged the total energy to <0.1 meV per atom. The unit cell dimensions were fixed in the initial cycles of optimization but then allowed to vary along with the coordinates. The convergence criteria were: 1 × 10^–8^ eV atom^–1^ (energy), 0.002 eV Å^–1^ (force), and 1 × 10^–4^ Å (displacement). The cell optimization was conducted in a soft compressibility scheme with fixed basis quality and a maximum stress of 0.01 GPa. Structures were visualized using both Mercury^18^ and Avogadro.^19^

### 2.3 Phonon Calculations

Following geometry optimization, vibrational frequencies were calculated in the harmonic approximation at the Γ-point and across the full Brillouin zone using the linear response method (i.e., density functional perturbation theory rather than the finite displacement method based on supercells).^20^ Anharmonicity and thermal expansion were neglected. For calculations across the Brillouin zone, the **q**-vector separation was set to 0.04 Å^–1^. The number of **q**-vectors (*N*) at this density was 3 for **1F**, 8 for **1,2F**, 14 for **1,4F**, 24 for **1,3,5F**, and 16 for **1,2,4,5F**. The frequencies of the acoustic modes at the Γ-point were within 0.06 cm^–1^ of 0 cm^–1^; all other frequencies were positive. Density of states (DoS) plots were calculated for all structures with the contributions of different atom types assessed at the Γ-point by use of partial density of states (PDoS) plots, this follows the method used by Kieslich et al.^12^

The effect of interpolation of the **q**-vector grid onto a finer grid spacing was investigated.^21^ For **1F**, the phonon interpolation grid was increased stepwise from 1 × 1 × 1 to 16 × 16 × 16, converging the calculated value of −*TS* at 150 K to within 0.01 kJ mol^–1^ (Table S3 in the Supporting Information). A 16 × 16 × 16 interpolation grid was used for all further calculations in this work.

Phonon calculations of increased computational expense were also applied to **1F**. The **q**-vector separation in this calculation was decreased from 0.04 to 0.03 Å^–1^, increasing *N* from 3 to 12. This led to a modestly increased occupation of the low energy vibrations but not such as to improve substantially the simulation of inelastic neutron scattering (INS) data (see Figures S1 and S2 in the Supporting Information). The value of −*TS* at 150 K also changed only modestly, from −10.31 to −10.42 kJ mol^–1^. The results of the calculations at the coarser spacing with interpolation were therefore accepted for all other derivatives in this study. Further information on convergence analysis of the phonon calculations can be found in Section 3 of the Supporting Information.

### 2.4 INS Measurements

Comparison of the experimental and simulated inelastic neutron scattering (INS) spectra was used to assess the accuracy of phonon calculations. Experimental INS data were measured at ISIS Neutron and Muon spallation source on the TOSCA indirect geometry spectrometer. Liquid samples for all compounds were obtained from Fluorochem. The samples were cold-ground under nitrogen before being loaded into cold aluminum cans and sealed.^22^ Spectra were recorded at ∼20 K, the base temperature of the instrument. Spectra for all samples were compared to simulated spectra obtained from phonon calculations by use of the program AbINS in Mantid.^23^ The temperatures used in the simulations were set to match the thermocouple readings taken from the experimental data files, and data from the front and the back detectors of TOSCA were merged.

### 2.5 Lattice and Intermolecular Interaction Energies

The lattice and intermolecular interaction energies for each optimized structure were calculated via the semiempirical Pixel method^24,25^ using the MrPixel interface.^26^ Gaussian-09^27^ was used for electron density calculations at the MP2 level of theory using the 6-31G** basis set. Molecular electron density was calculated on a grid of 0.08 × 0.08 × 0.08 Å^3^ and applied with a condensation level of 4 and a cluster radius of 14 Å. Component energies were calculated for Coulombic, polarization, dispersion, and repulsion interactions with total energy taken as the sum of all contributions. Contacts (labeled A, B...) within the first coordination sphere of **1F** and **1,2F** are listed along with their energies in Table 1. Data for all dimers are available in Table S5, with individual dimers shown in Figures S4–S8 all in the Supporting Information.

**Table 1:** Symmetry Unique Interactions within the First Coordination Spheres of **1F** and **1,2F**^a^

**structure**	**dimer**	**symmetry**	**contact type**	**centroid separation**	**Coulombic**	**polarization**	**dispersion**	**repulsion**	**total**	**total per molecule**
**1F**, **FACFAQ***P*4_3_2_1_2	A	*y* ± 1/2, −*x* + 5/2, *z* + 1/4; –*y* + 5/2, *x* ± 1/2, *z* – 1/4	H···π	4.759	–3.6	–1.8	–14.5	11.0	–8.9	–4.5
*Z* = 4	B	*x*, *y* ± 1, *z*; *x* ± 1, *y*, *z*	H···F, H···H	5.752	–1.7	–0.8	–9.1	4.5	–7.0	–3.5
Z′ = 1/2	C	*y* ± 1/2, −*x* + 3/2, *z* + 1/4; –*y* + 3/2, *x* ± 1/2, *z* – 1/4	H···F, H···H	6.542	–2.6	–1.2	–7.6	5.0	–6.4	–3.2
*U* = −52.0	D	*x* ± 1, *y* ± 1, *z*	H···F	8.134	–1.3	–0.2	–1.5	0.2	–2.8	–1.4
**1,2F, FACFOE***P*2_1_/*n*	A	–*x* + 3/2, *y* ± 1/2, −*z* + 3/2	H···π	4.686	–5.1	–1.8	–15.4	11.2	–11.1	–5.6
*Z* = 4	B	–*x* + 5/2, *y* ± 1/2, −*z* + 3/2	H···π	4.843	–4.1	–1.3	–12.6	7.4	–10.6	–5.3
Z′ = 1	C	*x* ± 1/2, −*y* + 1/2, *z* ± 1/2	H···F, H···F	6.782	–4.7	–1.1	–6.9	4.3	–8.5	–4.3
*U* = −49.8	D	–*x* + 2, −*y* + 1, −*z* + 1	F···F, F···F	5.864	–0.7	–0.8	–8.5	3.2	–6.8	–3.4
	E	–*x* + 2, −*y* + 1, −*z* + 2	H···H···H	5.954	–1.3	–0.7	–7.6	3.4	–6.2	–3.1
	F	*x*, *y* ± 1, *z*	H···F	6.007	–0.6	–0.9	–8.6	4.2	–5.9	–3.0
	G	*x* ± 1/2, −*y* + 3/2, *z* ± 1/2	H···F, H···H	6.935	–1.4	–0.7	–5.2	2.8	–4.5	–2.3
	H	–*x* + 2, – *y*, −*z* + 1	F···F	8.268	1.5	–0.2	–1.7	0.6	0.2	0.1

a All energies are given in kJ mol^–1^. *U* = lattice energy. Centroid separation distances are given in Å. Data for other derivatives and diagrams of each dimer are available in the SI (Table S5, Figures S4–S8)

### 2.6 Entropy Calculations

Vibrational entropies were calculated for all compounds from the frequency output of the phonon calculations across the Brillouin zone using routines available in CASTEP. Estimates of the entropy determined at the Γ-point, which are used below for illustrative purposes, were calculated as in Section 8 of the Supporting Information. Although the experimental structures were determined between 123 and 215 K, all entropies were calculated at 150 K to facilitate direct comparison.

### 2.7 Force Constants

Force constants were calculated for selected dimers within the first coordination sphere of **1,2F** in order to assess the relative deformability of C–H···F interactions. The dimers selected for **1,2F** were a C–H···π interaction (referred to below as contact A, see Figure S5 in the Supporting Information) and a bridged C–H···F interaction (contact C). The coordinates were taken from the DFT-optimized structure. For the purposes of comparison, the same procedure was applied to the H-bonded water dimer with a geometry taken from the Benchmark Energy and Geometry Database from data set S22.^28^

Calculation of the force constants was accomplished by evaluating the energy of a dimer as its geometry was distorted. Two methods were applied. In the first method, dimers were distorted along the eigenvectors of Γ-point phonons using the program ModeFollow.^29^ Modes were selected from within the low energy envelope of vibrations below 200 cm^–1^, which contribute most to entropy.^30,31^ For the bridged C–H···F interaction (contact C), three separate modes were selected; two modes provide the highest distortion of the C–H···F interaction and the third as the highest energy mode below the 200 cm^–1^ limit for external vibrations. These modes are numbered 16, 22, and 24 in Table S6 in the Supporting Information. The dimer of interest was isolated from the unit cell at each step along the distortion and the energy calculated for the isolated dimer (see below). The extent of distortion (*R*) for each mode was defined numerically as the average distance between atoms in their distorted state relative to their optimized positions. The same modes were studied for the C–H···π interaction (contact A) for comparison. Mode 16 for contact A and mode 24 for contact C produced very flat potentials, indicating that the energies of these modes are determined by the distortions of interactions with other molecules in the crystal structure; these modes were excluded from further analysis.

The second method of distortion followed the procedure of Carlucci and Gavezzotti^32^ This method was used for **1,2F** contacts A, C, and the water dimer. The interaction axis of each dimer was aligned along the Cartesian *z* axis using Avogadro.^19^ For both contacts A and C, the centroids of the aromatic rings were calculated to define this axis. For the water dimer, the axis was defined along the shortest H···O intermolecular distance. The separation distance of the two molecules were then linearly distorted along *z* by +1.0 and −1.0 Å from its optimized value in steps of 0.1 Å. Energies were calculated at each point.

The dimer energies in both sets of calculations were evaluated using symmetry adapted perturbation theory (SAPT) in the program Psi4.^33^ Convergence testing was carried out using contact C. All combinations of double, triple, and quadruple-ζ basis sets with the augmentation levels of August, July, and June from the “calendar” set (reducing backward in month for each diffuse shell removed from the calculation) were tested at all levels of SAPT truncation.^34^ On the basis of these tests, SAPT2 + 3 was selected with the aug-cc-pVDZ basis set for further calculations; further data are available in Section 4 of the SI.

The above calculations yielded potential curves *E*(*R*) for each interaction studied. The force constants, *k*^0^, for the dimers were calculated as in Carlucci and Gavezzotti^32^ using eq 1 after fitting polynomials to the potential curves; we found that order six polynomials yielded better fits than the fourth order set used in Carlucci et al.^32^
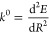
1

## 3 Results and Discussion

### 3.1 Intermolecular Interactions

Descriptions of the packing in all the fluorobenzene derivatives studied here (Figure 1) have been provided by Thalladi et al.^9^ The energies and centroid–centroid distances of the interactions formed in each structure, as determined by the Pixel method are given in Table 1 (**1F** and **1,2F**) and Table S5 in the Supporting Information (all derivatives). The structures contain 12 (**1,2,4,5F**), 13 (**1,2F**), or 14 (**1F**, **1,4F** and **1,3,5F**) molecules in the first coordination sphere, linked by C–H···F, C–H···H, and C–F···F contacts, which are in some cases bifurcated or bridged. The structures of **1F**, **1,2F**, and **1,4F** also contain C–H···π interactions, whereas those of **1,3,5F** and **1,2,4,5F** feature offset π···π interactions.

The lattice energies are similar, varying between −45.8 and −52.0 kJ mol^–1^. The modest values^25^ reflect the low melting points of these materials. The π···π or C–H···π interactions are more stabilizing than single, bridged, or bifurcated C–H···F interactions in all structures except **1,4F** where a bridged C–H···F interaction provides 0.5 kJ mol^–1^ greater stabilization than a C–H···π interaction.

The largest contribution to the C–H···F interactions is dispersion rather than electrostatics. This finding is in agreement with work by Sudheendranath et al. on α-fluorokeytones, where the dispersive term was found to outweigh the electrostatic term by 3–4 times and also with the survey of C, H, and F-containing structures by Gavezzotti and Lo Presti.^35^ Desiraju et al. have similarly reported that C–H···F interactions, which they describe as weak hydrogen bonds, have less pronounced electrostatic contributions.^36^

Although the aromatic nature of structures studied here contribute toward dispersion terms in these contacts, a search^1^ of the Cambridge Structural Database (CSD)^14^ reveals that average contact lengths for C–H···F bonds hardly vary for aromatic (2.512 Å) and nonaromatic (2.519 Å) structures (see Section 2 in the Supporting Information), indicating that formation of these contacts is not simply a feature of optimization of aromatic interactions.

The low energies of C–H···F interactions, as well as their lower or similar energies to π···π or C–H···π contacts, suggests that it is difficult on energy grounds alone to support their description^8,9^ as structure directing interactions. Nevertheless, their persistence in the crystal structures of organo-fluorine derivatives implies that they must make a significant stabilizing contribution.^4^ The aim of the following sections is to explore the role that entropy has in resolving this apparent paradox.

### 3.2 Phonon Calculations

#### 3.2.1 Inelastic Neutron Scattering Experiments

The entropy of crystalline solids is determined by access to low frequency vibrational (or phonon) energy levels. In disordered solids, there is also a contribution from alternative configurations, but since all structures studied here are ordered, this was not considered in the present study. In order to explore the role of entropy in the stabilization of crystal structures containing C–H···F interactions, it is thus necessary to calculate vibrational frequencies. In crystalline solids, internal vibrations occur within molecules and external vibrations occur between molecules. The relative phases of vibrational modes between unit cells defines different points in the Brillouin zone. The point where the vibrations of all unit cells are in phase defines the Γ-point; the opposite zone edge, which is given a variety of different symbols depending on crystal symmetry, occurs where vibrations in neighboring unit cells are out of phase.

Calculation of the vibrational properties of solids is computationally expensive, and approximations such as the assumption of harmonicity are usually invoked. This assumption was made in the present study. The effects of thermal expansion were also neglected. More elaborate models could be based on the quasi-harmonic approximation to account for thermal expansion or explicit inclusion of anharmonicity. Destabilizing (positive) contributions to free energy, namely, the increase in internal energy due to the occupation of vibrational modes as temperature increases and zero-point energy, were also neglected. These quantities can differ significantly in crystal structures with very strong intermolecular interactions,^30^ but differences between polymorphs are usually very small;^11^ further comments are available in Section 9 of the Supporting Information.

**Figure 2:**
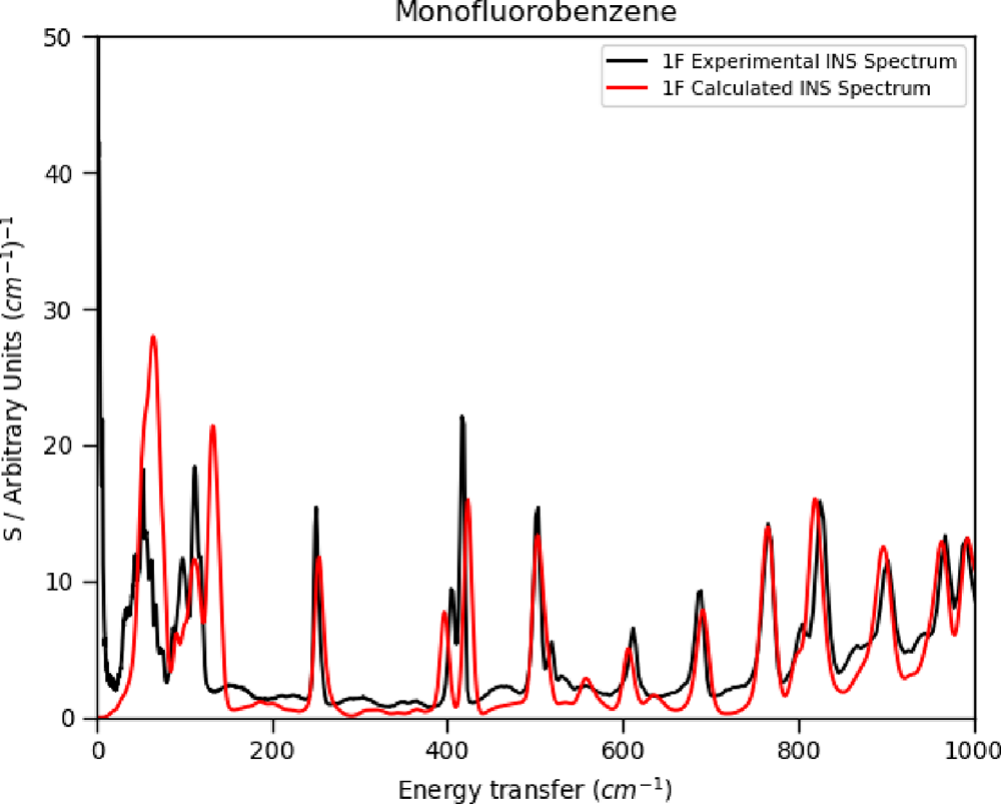
Experimental and simulated INS spectra for **1F**. Spectra for other compounds and larger spectral ranges can be found in Figures S9–S13 of the Supporting Information.

Inelastic neutron scattering data, which provides very useful benchmarking data for phonon spectra calculated by periodic DFT,^23^ were collected in order to determine the impact of our assumptions on the accuracy of the phonon calculations. Calculated and experimental INS spectra in the range 0–1000 cm^–1^ are presented in Figure 2 for **1F**. Spectra for all structures and spectra over larger ranges can be found in Figures S9–S13 in the Supporting Information. The intensities of measured INS spectra depend on experimental parameters such as the sample volume and incident beam intensity as well as the characteristics of the vibrations, and the simulated intensities were scaled visually to match the peaks near 1000 cm^–1^. In all cases, the simulated spectra show a level of agreement with the experimental data that is typical in comparable studies employing periodic DFT and the harmonic approximation.^37^ The frequencies and intensities of the internal modes are well reproduced. The external modes below 200 cm^–1^ show small frequency offsets which reflect the assumptions described above and which tend to lead to overestimation of the frequencies.^38^ Nevertheless, the essential features of the INS spectra below 200 cm^–1^ have been reproduced. Inclusion of thermal expansion, for example, by use of a quasi-harmonic model,^39^ would be expected to decrease the frequencies,^40^ while inclusion of anharmonic effects usually, though not always, also leads to a decrease in calculated frequencies when compared to the harmonic approximation.^41^

#### 3.2.2 Entropy Calculations

The significance of the offsets between the experimental and DFT simulations in the low frequency vibrational modes can be assessed by comparison of the calculated entropies with experimental values, which are available from heat capacity (*C*_*p*_) data for **1F**, **1,2F**, and **1,2,4,5F**.^42−44^

The values of −*TS* (*T* = temperature, *S* = entropy) terms for all five compounds at 150 K are listed in Table 2 alongside experimental values where available. The value of 150 K used here and below is representative of the temperatures of the experimental structure determinations. The experimental and simulated values of −*TS* in Table 2 all agree to within about 2 kJ mol^–1^, the underestimation in the simulated values being consistent with the slight overestimation in the positions of the low frequency bands shown in Figure 2. The calculations also recover the increasing trend with fluorine content.

**Table 2:** Values of −*TS* at 150 K for Fluorobenzenes Calculated Using Periodic DFT at the Γ-Point and Across the Brillouin Zone Compared to Available Data Determined Experimentally from Heat Capacity Measurements; All Values in kJ mol^–1^

	Γ phonon	full phonon	experimental
**1F**	–8.63	–10.31	–12.42^42^
**1,2F**	–9.99	–11.73	–13.72^43^
**1,4F**	–8.58	–12.33	
**1,3,5F**	–9.83	–13.85	
**1,2,4,5F**	–11.40	–15.05	–15.77^44^

Thus, although the agreement is not perfect, the INS and entropy benchmarking data indicate that the level of theory applied in this work is suitable for the purpose of estimating the magnitude of entropy effects in weakly bound crystal structures such as those of fluorobenzenes.

#### 3.2.3 Partial Density of States Calculations

The values of −10 to −15 kJ mol^–1^ for the −*TS* contribution to the stabilization of fluorobenzenes at 150 K shown in Table 2 is comparable to the enthalpic stabilization afforded by the N–H···N and O–H···O hydrogen bonds in the crystal structure of ammonia (−9.3 kJ mol^–1^) and the optimized water dimer (−20.6 kJ mol^–1^).^45^ But what contribution is made by C–H···F interactions toward this stabilization?

**Figure 3:**
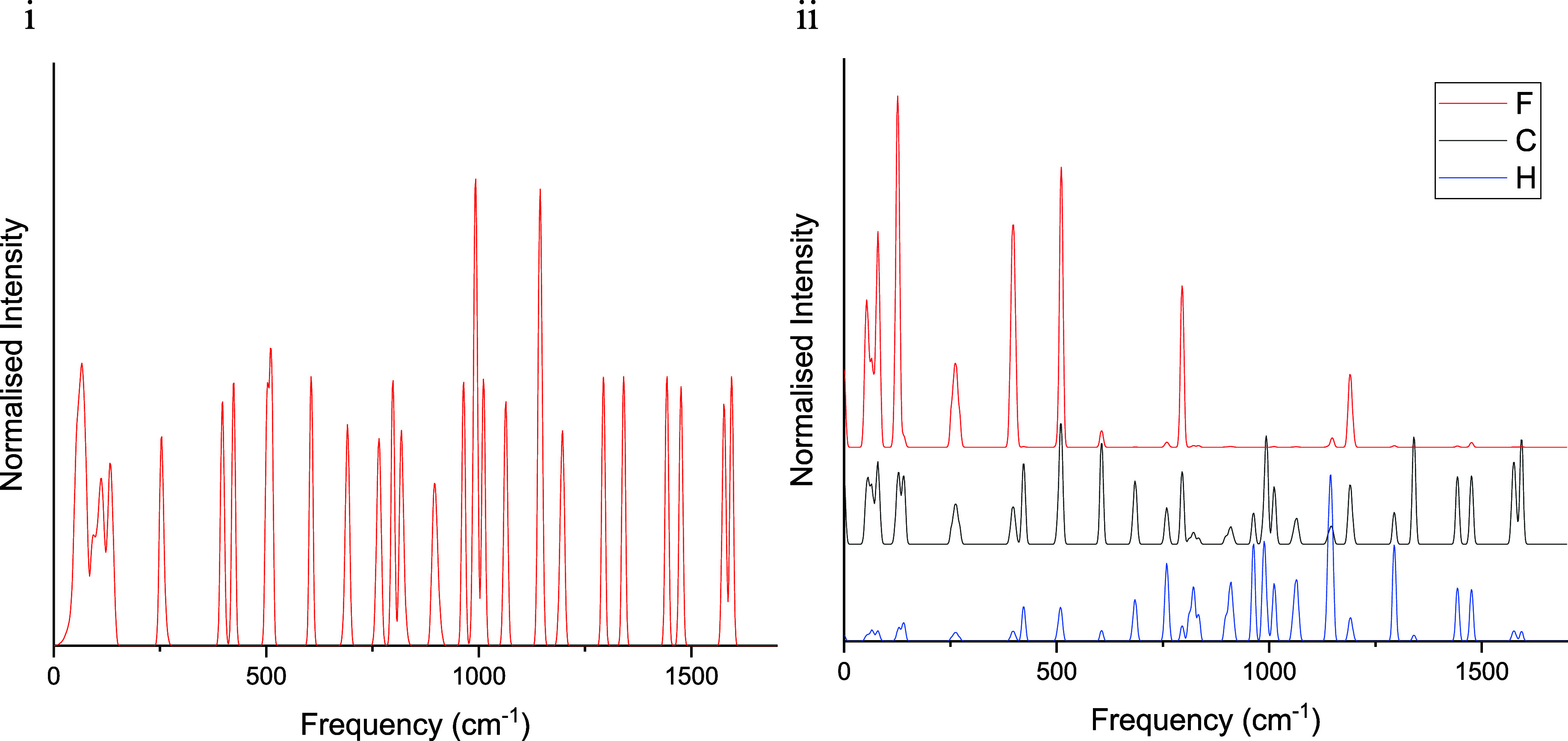
(i) Density of states for **1F** in the range 0–1700 cm^–1^. (ii) Partial density of states per atom type per atom for **1F**. Red is fluorine, black is carbon and blue is hydrogen. Data for individual atom types have been offset for clarity. Plots for all other structures and larger spectral ranges are available in Figures S14–S23 in the Supporting Information.

The phonon density of states, shown in Figure 3i for **1F**, were decomposed into contributions from each atom type at the Γ-point; plotting these partial density of states reveals how individual modes are distributed over different atoms.^12^ The advantage of using the Γ-point is that the contribution of entropy by bands of increasing frequency is easily calculated by hand (Section 8 and Table S6 of the Supporting Information), providing useful insight into the importance of low energy vibrations; note that the entropy calculated in this way is underestimated because the contributions of acoustic phonons are missing (see Table 2).

The partial density of states for **1F** are shown for F, C, and H in Figure 3ii, with the remaining compounds shown in Figures S19–S23 in the Supporting Information. For all structures, the fluorine atoms contribute significantly more per atom to the modes below 200 cm^–1^ than C or H, as indicated by the higher intensity of the peaks within this range for the fluorine atoms.

#### 3.2.4 Force Constant Calculations

The thermodynamic contribution made by an intermolecular interaction to the stability of a phase depends on the characteristics of its potential. The enthalpic significance depends on the depth of the potential. The entropic significance depends on the shape of the potential at its minimum. A low curvature at the potential minimum is indicative of a deformable interaction with a low force constant (eq 1). An interaction with a low force constant is associated with a low vibrational frequency and hence a high entropy. However, vibrational frequencies also depend on mass, and low frequencies are also associated with vibrations, which involve heavy atoms.

The partial density of states analysis indicates that the motions of the fluorine atoms contribute most to the lowest energy vibrations in fluorobenzenes and therefore contribute most to entropy. The question we address in the following section is whether this is simply because fluorine is the heaviest atom present or whether the low energy of vibrations involving fluorine can also be ascribed to the low force constants, i.e., the deformability, of C–H···F interactions. Answering this question requires explicit calculation of the potentials of intermolecular interactions involving C–H···F contacts, along with those of other classes of interaction for the purposes of comparison.

**Figure 4:**
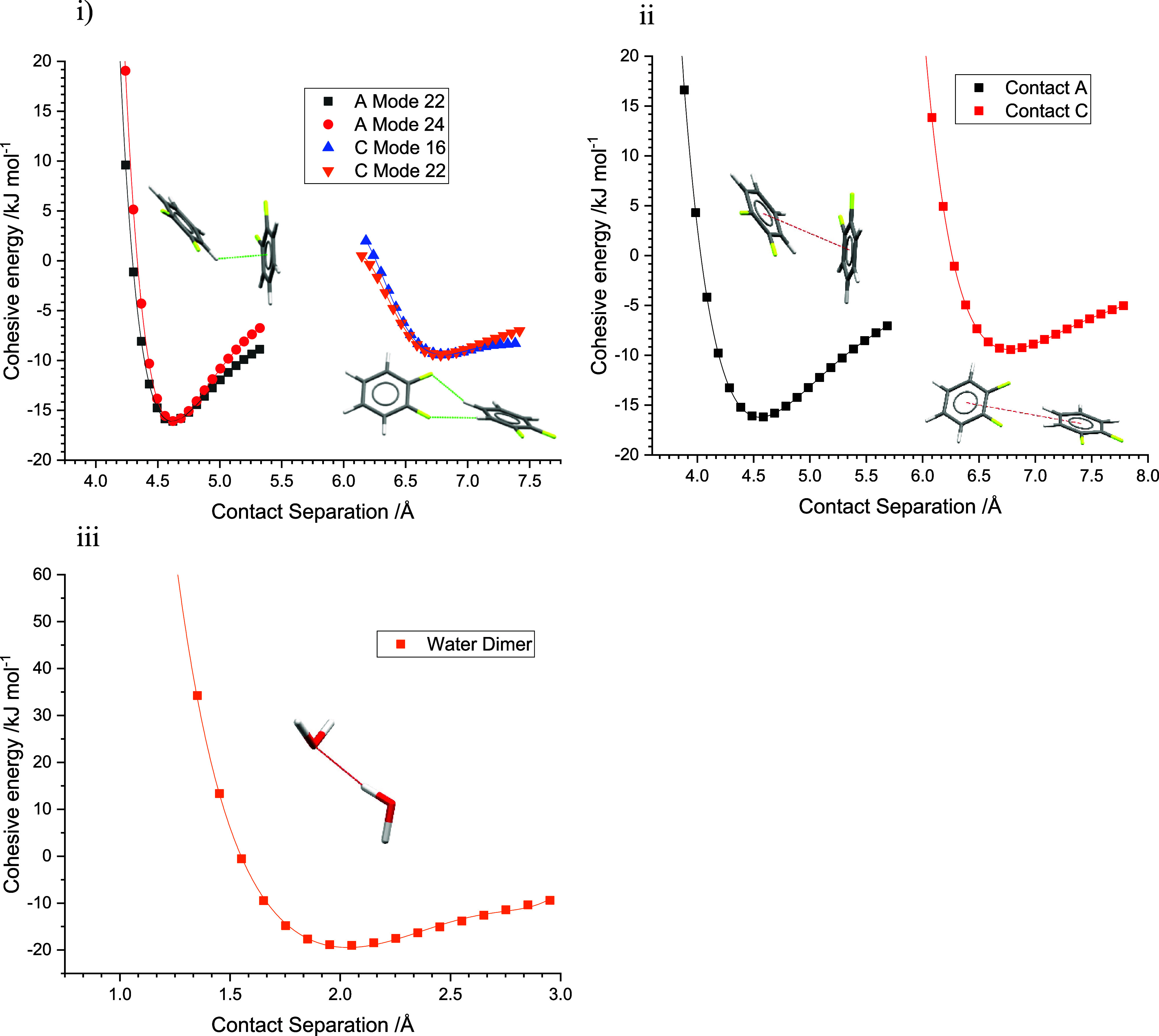
Energy potentials for (i) distortions along phonon modes for contacts A and C for **1,2F** (method 1). (ii) Distortions of centroid–centroid distances for contact A and C in **1,2F** (method 2). (iii) Distortion of the O···H distance in the water dimer (method 2). Red dashed lines in (ii) and (iii) represent defined interaction axes for method 2.

Potentials were calculated for contacts selected from the crystal structure of **1,2F**. Contact C (Table 1, Figure S5) is formed across an inversion center and contains two bridged C–H···F interactions; for comparison, the same calculations were carried out for the C–H···π interaction, contact A. The force constants for both contacts were calculated by two methods as described in Section 2.7. For comparison, the second method was applied to the isolated water dimer; the same method has been applied to the pentafluoroiodobenzene-pyrazine C–I···N halogen bond in ref (32). The potentials and force constants are shown in Figure 4 and Table 3, which also reports separation distances and energies from the optimized structure alongside equilibrium distances and cohesive energies.

**Table 3:** Equilibrium Distances (*R*_opt_) and Energies (*E*_opt_) from the DFT-Optimized Structures and the Minimum Distances (*R*^0^), Energies (*E*^0^), and Force Constants (*k*^0^) for Selected Contacts in **1,2F** and Water from the Fitted Potentials^a^

**contact/mode**	***R*_opt_**	***E*_opt_**	***R*^0^**	***E*^0^**	***k*^0^**
method 1					
A/22	4.686	–15.813	4.615	–16.097	136.6
A/24	“	“	4.625	–16.103	188.2
average			4.620	–16.100	162.4
C/16	6.782	–9.421	6.806	–9.445	38.5
C/22	“	“	6.787	–9.440	43.0
average			6.797	–9.443	40.8
method 2					
A	4.686	–15.813	4.556	–16.211	55.6
C	6.782	–9.421	6.772	–9.421	31.7
Water	1.952	–18.867	2.036	–19.455	94.2
FBz-I^32^			3.17	–9.9	97

a Data for the C–I···N interaction in the pentafluoroiodobenzene-pyrazine dimer (**FBz-I**) taken from Carlucci and Gavezzotti are listed for comparison.^32^ Distances are in Å, energies in kJ mol^–1^ and force constants in kJ mol^–1^ Å^–2^.

For the **1,2F** modes, the minimum of the fitted polynomial was slightly shorter than the separation within the optimized structure, a reflection of interaction distances in crystal structures being the result of a competition between the pushes and pulls of all contacts present, so that none adopt exactly the same geometry as an isolated dimer.^46^ The C–H···π interaction (A) is stronger than the bridged C–H···F interaction (C) with equilibrium energies of −15.8 and −9.4 kJ mol^–1^, respectively (Figure 4i,ii; the corresponding values in the crystal structure calculated by the Pixel method are −11.1 and −8.5 kJ mol^–1^). The potentials for contact C in Figure 4i,ii are distinctly shallower than for contact A, producing substantially lower force constants. The average of the of the force constants calculated by method 1 are 162.4 and 40.8 kJ mol^–1^ Å^–2^ for contacts A and C, respectively. Although the results of method 2 are different (they correspond to different distortions), they present a consistent picture: 55.6 and 31.7 kJ mol^–1^ Å^–2^ for A and C, respectively. Comparative figures for the hydrogen and halogen bonded dimers in Table 3 are both of the order of 100 kJ mol^–1^ Å^–2^.

The energies of the interactions discussed here lie between about 5 and 20 kJ mol^–1^; the energetic differences are accompanied by a distribution in the range of values for the force constants, with those for C–H···F interactions being significantly lower than those of other contacts. These data indicate that the entropic contribution of C–H···F interactions stems not only from the mass of the fluorine atom but also their deformability.

## 4 Conclusions

The current work was inspired by a comment made by Taylor in a paper entitled *It Isn’t, It Is: The C–H···X (X = O, N, F, Cl) Interaction Really Is Significant in Crystal Packing*.^4^ The final paragraph of that paper reads: *It took me 30 years to be persuaded that C–H···F–C... contacts matter, but I remain of this view. Whether this belief is accepted by others or not, one thing is clear: any explanation of the crystal packing of the structures... must account for the fact that they contain many more X···H interactions than would be expected by chance.*

Although C–H···F interactions are weak, they occur frequently, 3.5 times more likely than would be expected by chance alone. Taylor reasoned that *The simplest explanation is that X···H* (X being a halogen) *interactions are sufficiently favorable in themselves to ensure their over-representation in crystal structures*. The hypothesis explored in the present contribution is that the favorable feature of C–H···F interactions lies in their contribution to entropy.

The vibrational, enthalpic, and entropic properties of five fluorobenzene derivatives have been investigated using periodic DFT, SAPT, and Pixel calculations. The phonon calculations, which were carried out in the harmonic approximation and neglected the effects of thermal expansion, nevertheless adequately reproduced the experimental entropies, determined from heat capacity data and INS spectra. The molecule–molecule energies associated with individual C–H···F interactions lie between −5 and −10 kJ mol^–1^. The interactions are largely dispersive, rather than electrostatic in character. The total contribution of entropy is between −10 and −15 kJ mol^–1^ at 150 K, more akin to the enthalpic stabilization made by a moderate hydrogen bond.

Entropy in ordered, crystalline solids is derived from access to low energy vibrational modes. Each mode is distributed over many different interactions in solids, and one of the difficulties encountered in the work described here has been to assess the entropic contribution of interactions involving C–H···F contacts. One technique has been to examine partial phonon density of states plots, which show that the lowest energy modes in fluorobenzenes are dominated by the motions of fluorine atoms. Another has been to investigate the properties of the potentials of dimers mediated by C–H···F interactions and to compare them with those for other classes of contact. This analysis showed that the low vibrational energies associated with C–H···F interactions reflect the relatively high mass of the halogen atom but also their deformability, with potentials which are flatter than other contacts such as C–H···π interactions and hydrogen and halogen bonds. The prevalence C–H···F interactions thus stems from a combination of the entropic advantage conferred by these two features as well as from the energies of the interactions themselves.

Taylor’s study shows that many weak interactions form randomly and tend not to be structure directing. For example, enthalpically weak C–O···C and C–N···C interactions were found to both occur 0.5 times less than would be expected by chance. However, C–H···F interactions do matter, the results presented here implying that entropy can have significant role to play in structures dominated by enthalpically weak interactions. The results of this work suggest that entropy can be an important consideration when interpreting the structural significance of weak interactions.

## Supporting Information Available

Results for all derivatives shown in Table 1 where illustrative data have been included in the main text: details of intermolecular interactions formed in the first coordination sphere of each structure along with the results of Pixel calculations (Section 5); comparison of experimental and simulated INS spectra (Section 6); phonon density of states data (Sections 7 and 8); comparison of optimized and experimental unit cell dimensions (Section 1) further details on the convergence of phonon and SAPT calculations are given in Sections 3 and 4; illustrative data on calculations for **1,2F** are available in Section 8. Section 2 lists criteria and results of a search of the Cambridge Structural Database for aromatic and nonaromatic fluorides. Section 9 outlines the justification for neglect of internal thermal energy and zero-point energy in the analysis presented (PDF)

## Footnotes

1 Relevant interactions were defined in the search as those shorter than the sum of the contributing van der Waals radii. Database entries without coordinates, *R*-factors above 5%, or those containing disorder, errors, powder data or ions were filtered out. Hydrogen bond lengths were normalized to neutron values. Searches were conducted on the CSD version 2023.2.0.
