# Supplementary material for: The Advantages of Flexibility: The Role of Entropy in Crystal Structures Containing C–H···F Interactions
Source: Cryst Growth Des. 2024 Feb 19;24(5):2217–25. doi: 10.1021/acs.cgd.4c00042 (PMC10921377; doi:10.1021/acs.cgd.4c00042)
Supplement: Supplementary file 1 — cg4c00042_si_001.pdf [file cg4c00042_si_001.pdf]

SUPPLEMENTARY MATERIAL

The Advantages of Flexibility: The Role of Entropy in Crystal Structures Containing C—H···F Interactions.

Cameron J. G. Wilson,[a] Jan Plesniar,[a] Heike Kuhn,[a] Jeff Armstrong,[b] Peter A. Wood[c] and Simon Parsons[1a]

a. Centre for Science at Extreme Conditions, School of Chemistry, The University of Edinburgh, King's Buildings, West Mains Road, Edinburgh, EH9 3FJ, UK.

b. ISIS Facility, STFC, Rutherford Appleton Laboratory, Chilton, Didcot, Oxfordshire, OX11 0QX, UK.

c. The Cambridge Crystallographic Data Centre, 12 Union Road, Cambridge, CB2 1EZ, UK

Abstract

Molecular crystal structures are often interpreted in terms of strong, structure directing, intermolecular interactions, especially those with distinct geometric signatures such as H-bonds or π-stacking interactions. Other interactions, whether they are weak or lack a characteristic geometry, can be overlooked. We show that although the cumulative effect of weak interactions is significant, their deformability also leads to occupation of low energy vibrational energy levels which provides an

[1] Corresponding author contact: S.Parsons@ed.ac.uk

additional stabilizing entropic contribution. The entropies of five fluorobenzene derivatives have been calculated by periodic DFT calculations to assess the entropic influence of C—H···F interactions in stabilizing their crystal structures. Calculations reproduce inelastic neutron scattering data and experimental entropies from heat capacity measurements. C—H···F contacts are shown to have force constants which are around half of those of more familiar interactions such as hydrogen bonds, halogen bonds and C—H···π interactions. This feature, in combination with the relatively high mass of F, means that the lowest energy vibrations in crystalline fluorobenzenes are dominated by C—H···F contributions. C—H···F contacts occur much more frequently than would be expected from their enthalpic contributions alone, but at 150 K the stabilizing contribution of entropy provides, at −10 to −15 kJ mol^{-1}, a similar level of stabilization to the N—H···N hydrogen bond in ammonia and O—H···O hydrogen bond in water.

Contents

1. Cell optimisation 3
2. Comparison of aromatic H…F interactions 4
3. Convergence of phonon calculations 5
4. SAPT Calculation convergence 7
5. Diagrams and energies of dimers for all structures 9
6. Inelastic Neutron Scattering plots 12
7. Density of states plots 15
8. Partial density of states plots 18
9. Justification for the neglect of *U* and zero-point energy 26

1. Cell optimisation

To provide a suitable level of geometrical convergence for the phonon calculations, experimental crystal structures were geometry-optimized as described in the main text. The optimized unit cell dimensions are compared to experimental values in Table S1. The structures are all monoclinic except for **1F**, which is tetragonal.

Table S1: Comparison between the experimental and optimised unit cells for all structures studied.

	FACFAQ (**1F**)		FACFOE(**1,2F**)		FACGEV(**1,4F**)		PVVAWA01(**1,3,5F**)		FACJAU(**1,2,4,5F**)	
	Exp	Opt	Exp	Opt	Exp	Opt	Exp	Opt	Exp	Opt
a/Å	5.799(2)	5.7515	7.4806(11)	7.3941	5.809(2)	5.7979	6.160(2)	6.0959	4.4719(11)	4.4285
b/Å	5.799(2)	5.7515	5.9608(9)	6.0073	6.530(2)	6.3318	11.909(3)	11.9534	10.285(2)	10.2540
c/Å	14.530(7)	14.4875	11.725(2)	11.8097	7.190(2)	7.0582	7.504(2)	7.4034	6.342(2)	6.4289
β/°	90.00	90.00	103.815(11)	103.9730	101.89(2)	101.9271	95.47(2)	95.1573	107.97(2)	107.8677

2. Comparison of aromatic H…F interactions

Searches of the Cambridge Structural Database[1] were conducted to investigate whether the formation of C—H···F interactions is simply a consequence of the optimization of aromatic interactions. Relevant interactions were defined as those shorter than the sum of the contributing van der Waals radii. Data without 3D coordinates, R-factors above 5%, containing disorder, errors, powder data or ions were filtered out of searches and hydrogen bond lengths were normalised to neutron values. Searches were conducted on the CSD 2023.2.0 dataset. The average distances shown in Table S2 no significant systematic difference between the lengths of C—H···F contacts in aromatic and non-aromatic systems.

Table S2: A comparison of the average bond length of intermolecular H···F interactions in the CSD and the aromatic and non-aromatic subsets of this.

Interaction		Length /Å
1, All Intermolecular	X—H…F—X	2.51750
2, Aromatic	Ar—H…F—Ar	2.51252
3, Non-Aromatic	1 and not 2	2.51933

3. Convergence of phonon calculations

Phonon frequencies for all structures were calculated in the harmonic approximation using the linear response method implemented in CASTEP (as opposed to the finite displacement method based on supercells) using a grid of **q**-points with a spacing of 0.04 Å^{-1}. The results were then interpolated onto a finer grid of 16 x 16 x 16 **q**-points. [Note: **q**-points define the positions in reciprocal space where phonon frequencies are calculated.] Our approach was based on the following convergence tests.

Calculations of increased computational expense were applied to monofluorobenzene, **1F**. An increase in the density of **q**-points from 0.04 Å^{-1} to 0.03 Å^{-1}, yielding an increase from 3 to 12 **q**-points, was applied. Levels of interpolation from 1 x 1 x 1 to 16 x 16 x 16 were then applied to the results obtained at 0.04 and 0.03 Å^{-1}; for even-numbered interpolations (*e.g.* 2 x 2 x 2), a grid shift was applied to include the Γ-point in the interpolation grid. The value of *TS* at 150 K was calculated for each interpolated **q**-point set. The results are tabulated in Table S3.

The value of *TS* converged with a grid of $16 \times 16 \times 16$ to within 0.01 kJ mol^{-1} for calculations based on the 0.04 and 0.03 Å^{-1} grids. At this level of interpolation, the difference between the values of *TS* for the two sets of calculations was of the order of 0.1 kJ mol^{-1}.

A comparison of the DoS and calculated INS spectra for the 0.04 and 0.03 Å^{-1} **q**-point sets at the highest level of interpolation are provided in Figure S1 and Figure S2 respectively; note that the integrals of both DoS plots are normalised to three times the number of atoms in one unit cell. The differences are very small.

The minor impacts on calculated entropies, DoS plots and INS spectra led us to apply a **q**-point spacing of 0.04 Å^{-1} interpolated to 16 x 16 x 16 for all further calculations.

Table S3: Effect of **q**-point density on the value of *TS* at 150 K for **1F**.

Interpolation level	3 **q**-points (0.04 Å^{-1})	12 **q**-points (0.03 Å^{-1})
$1 \times 1 \times 1$	8.62568	8.62568
$2 \times 2 \times 2$	10.23244	10.48119
$3 \times 3 \times 3$	10.21952	10.34219
$4 \times 4 \times 4$	10.30388	10.42043
$5 \times 5 \times 5$	10.28744	10.39312
$6 \times 6 \times 6$	10.30705	10.41631
$7 \times 7 \times 7$	10.30138	10.40712
$8 \times 8 \times 8$	10.31065	10.41693
$9 \times 9 \times 9$	10.30564	10.41235
$10 \times 10 \times 10$	10.30979	10.41631
$11 \times 11 \times 11$	10.30758	10.41490
$12 \times 12 \times 12$	10.31058	10.41637
$13 \times 13 \times 13$	10.30893	10.41499
$14 \times 14 \times 14$	10.31087	10.41852
$15 \times 15 \times 15$	10.30932	10.41554
$16 \times 16 \times 16$	10.31041	10.41719

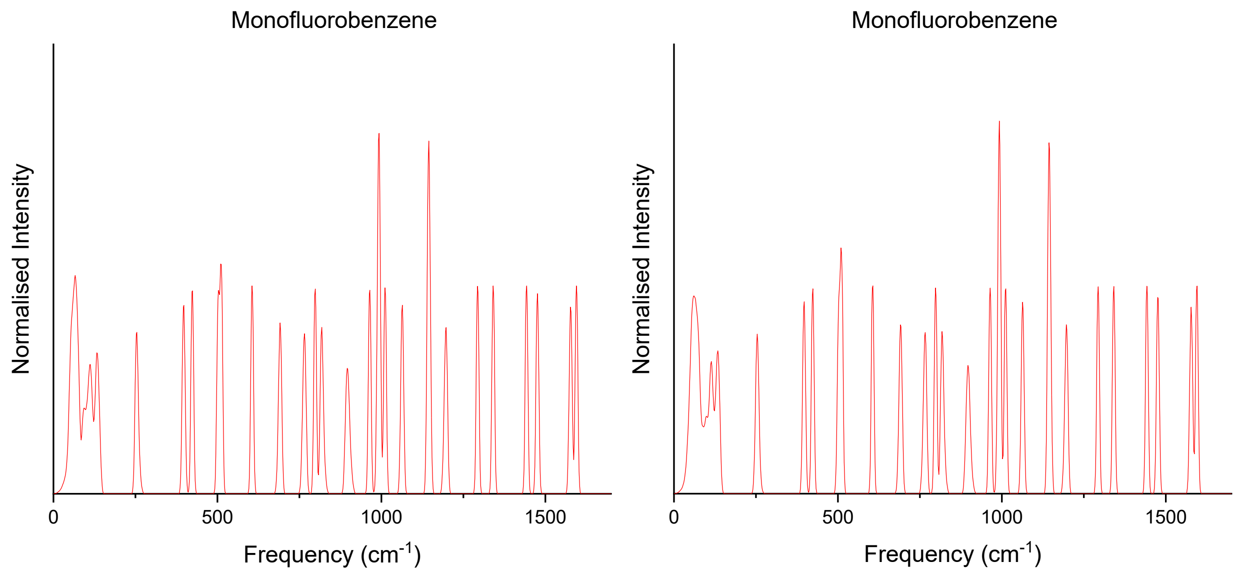

Figure S1: The impacts of q-point density on DoS plots of **1F**. Left: 3 **q**-points (0.04 Å^{-1} spacing), right: 12 **q**-points (0.03 Å^{-1} spacing) interpolated to a 16 × 16 × 16 grid.

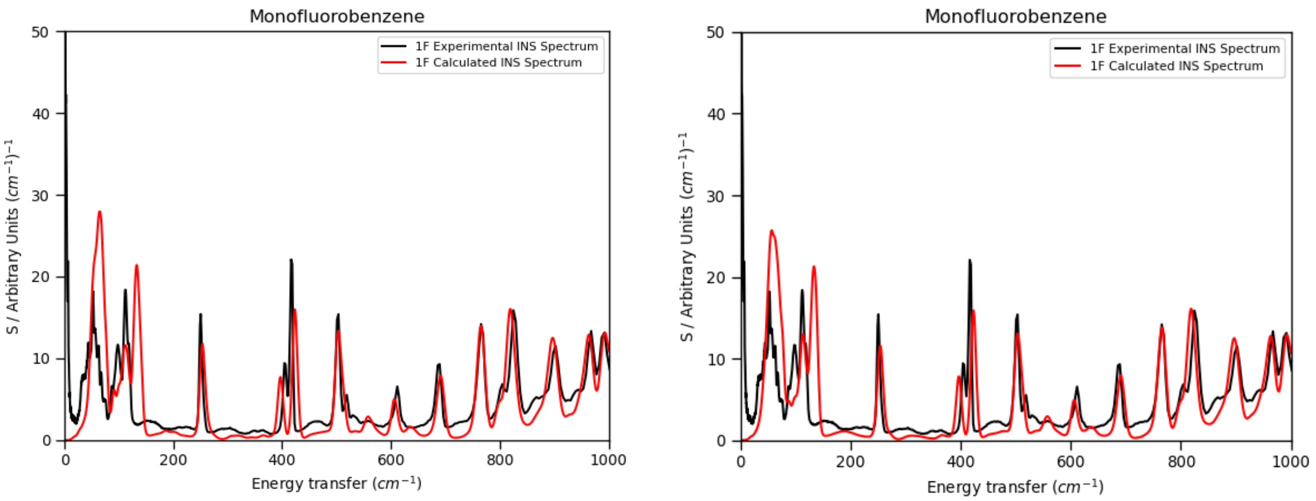

Figure S2: The impacts of q-point density on INS spectra of **1F**. Left: 3 **q**-points (0.04 Å^{-1} spacing), right: 12 **q**-points (0.03 Å^{-1} spacing) interpolated to a 16 × 16 × 16 grid.

4. SAPT Calculation convergence

Dimer energies were calculated for mapping potentials using symmetry adapted perturbation theory (SAPT) through the program Psi4.[2] Convergence testing of the most appropriate level of theory for these calculations was completed using a dimer of 1,2-difluorobenzene, **1,2F**, contact C, a bridged C—H···F interaction (below). Combinations of the double, triple and quadruple zeta basis sets with the augmentation levels of August, July and June from the 'calendar' set (reducing backwards in month for each diffuse shell removed from the calculation) were tested at all levels of SAPT truncation.[3] The results are shown in Table S4. The wall times refer to the SAPT2+3 calculations, which were given 150 GB of memory and 30 processors. Convergence of SAPT energies against time taken for the calculations is shown in Figure S3.

SAPT2+3 was considered the most reliable for all computations. From Table S4 it is clear that, especially the for the lower basis sets, the augmentation level of the calculation has a comparatively modest impact on wall times whilst giving significant improvements to calculated energies. Increases in basis set quality however are very computationally expensive. For the august augmentation level, the shift from a double zeta to quadruple zeta basis set would change the calculation time of a 21-point potential well from ~1 day to ~85 days. From Figure S3 an initial significant improvement to the energy quickly flattens to an area where modest energy improvements require multiple hour increases in wall time. It is from the beginning of this flattening that the aug-cc-pVDZ basis was chosen.

Table S4: Wall times and calculated energies for different levels of SAPT theory.

Augmentation level	Basis set	Wall time /s	Wall time /h	SAPT0	SAPT2	SAPT2+	SAPT2+(3)	SAPT2+3
aug-cc-pVXZ	aDZ	4159	1.155277778	-9.03150157	-8.31043818	-7.99142424	-7.43396411	-7.65941191
	aTZ	44983	12.49527778	-9.55937917	-9.17881487	-8.80731815	-8.11903590	-8.40289603
	aQZ	347775	96.60416667	-9.73965335	-9.48913342	-9.03262549	-8.27017400	-8.58993156
heavy-aug- OR	haDZ	2401	0.666944444	-8.79201228	-8.06491298	-7.66813311	-7.14694053	-7.36329872
jul-cc-pVXZ	haTZ	36462	10.12833333	-9.44328157	-9.06042716	-8.64521165	-7.98983586	-8.26182458
	haQZ	324877	90.24361111	-9.67965261	-9.42998841	-8.95197416	-8.21810102	-8.52649965
jun-cc-pVXZ	jaDZ	1441	0.400277778	-6.14861685	-5.55568244	-5.03569801	-4.62352795	-4.80044752
	jaTZ	23925	6.645833333	-9.14506180	-8.76504080	-8.24003207	-7.64289891	-7.89102228
	jaQZ	214196	59.49888889	-9.63292755	-9.38230719	-8.88186333	-8.16804894	-8.46803956

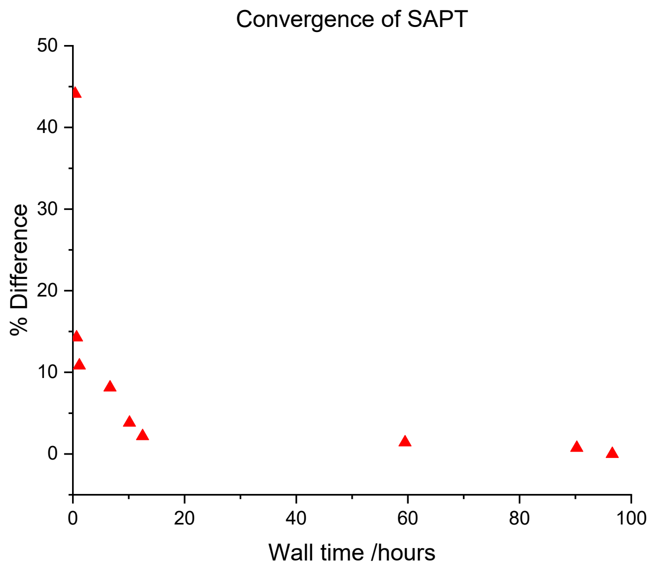

Figure S3: Convergence of relative SAPT energies. The y-axis has been extended towards negative % differences for clarity of values which would otherwise lie very close to the x-axis.

5. Diagrams and energies of dimers for all structures

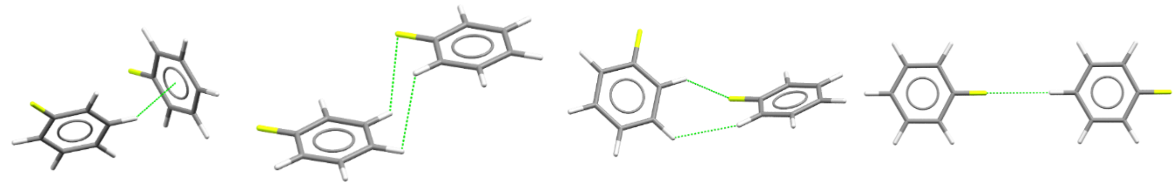

A B C D

Figure S4: The symmetry inequivalent dimers within **1F** (monofluorobenzene), FACFAQ.

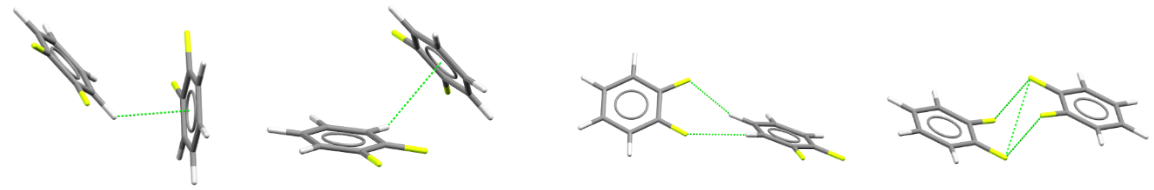

A B C D

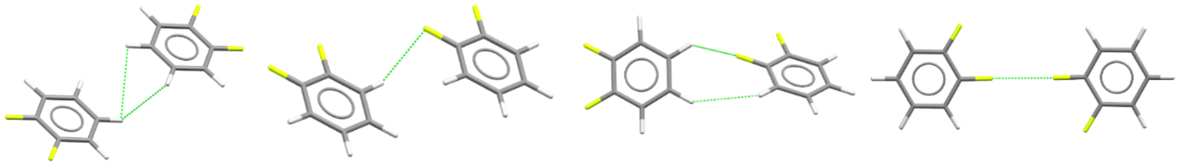

E F G H

Figure S5: The symmetry inequivalent dimers within **1,2F** (1,2-difluorobenzene), FACFOE.

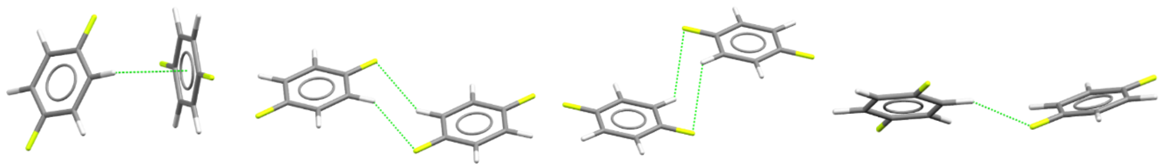

A B C D

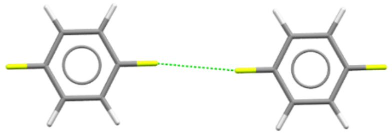

E

Figure S6: The symmetry inequivalent dimers within **1,4F** (1,4-difluorobenzene), FACGEV.

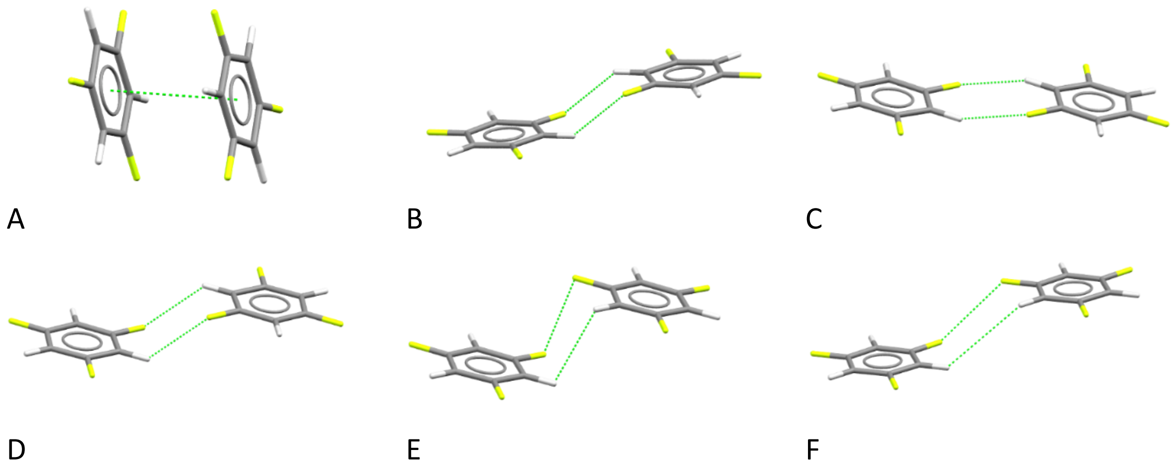

Figure S7: The symmetry inequivalent dimers within **1,3,5F** (1,3,5-trifluorobenzene), PVVAWA01.

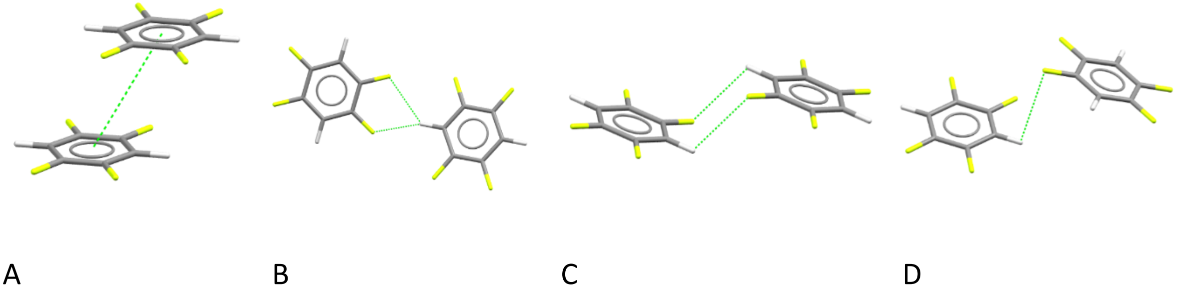

Figure S8: The symmetry inequivalent dimers within **1,2,4,5F** (1,2,4,5-tetrafluorobenzene), FACJAU.

Table S5: Symmetry unique interactions within the first coordination spheres of fluorobenzenes. All energies are given in kJ mol^{-1}. *U* = lattice energy.

Structure	Dimer	Symmetry	Contact Type	Centroid separation	Coulombic	Polarization	Dispersion	Repulsion	Total	Total per molecule
**1F**, **FACFAQ**<br>$P4_32_12$	A	$y\pm1/2, -x+5/2, z+1/4$<br>$-y+5/2, x\pm1/2, z-1/4$	H···π	4.759	−3.6	−1.8	−14.5	11.0	−8.9	−4.5
Z = 4,	B	$x, y\pm1, z; x\pm1, y, z$	H···F, H···H	5.752	−1.7	−0.8	−9.1	4.5	−7.0	−3.5
Z' = ½	C	$y\pm1/2, -x+3/2, z+1/4$<br>$-y+3/2, x\pm1/2, z-1/4$	H···F, H···H	6.542	−2.6	−1.2	−7.6	5.0	−6.4	−3.2
U = −52.0	D	$x\pm1, y\pm1, z$	H···F	8.134	−1.3	−0.2	−1.5	0.2	−2.8	−1.4
**1,2F, FACFOE**	A	$-x+3/2, y\pm1/2, -z+3/2$	H···π	4.686	−5.1	−1.8	−15.4	11.2	−11.1	−5.6
$P2_1/n$	B	$-x+5/2, y\pm1/2, -z+3/2$	H···π	4.843	−4.1	−1.3	−12.6	7.4	−10.6	−5.3
Z = 4	C	$x\pm1/2, -y+1/2, z\pm1/2$	H···F, H···F	6.782	−4.7	−1.1	−6.9	4.3	−8.5	−4.3
Z' = 1	D	$-x+2, -y+1, -z+1$	F···F, F···F	5.864	−0.7	−0.8	−8.5	3.2	−6.8	−3.4
U = −49.8	E	$-x+2, -y+1, -z+2$	H···H···H	5.954	−1.3	−0.7	−7.6	3.4	−6.2	−3.1
	F	$x, y\pm1, z$	H···F	6.007	−0.6	−0.9	−8.6	4.2	−5.9	−3.0
	G	$x\pm1/2, -y+3/2, z\pm1/2$	H···F, H···H	6.935	−1.4	−0.7	−5.2	2.8	−4.5	−2.3
	H	$-x+2, -y, -z+1$	F···F	8.268	1.5	−0.2	−1.7	0.6	0.2	0.1
**1,4F, FACGEV**<br>$P2_1/c$	A	$-x+1, y\pm1/2, -z+3/2$<br>$-x+1, y\pm1/2, -z+5/2$	H···π	4.741	−3.3	−1.3	−13.9	9.2	−9.4	−4.7
Z = 2,	B	$x, y\pm1, z$	H···F, H···F	6.332	−5.3	−1.3	−8.6	5.0	−10.3	−5.2
Z' = ½	C	$x\pm1, y, z$	H···F, H···F	5.798	−2.8	−0.9	−9.3	4.4	−8.5	−4.3
U = −50.0	D	$-x, y\pm1/2, -z+3/2$<br>$-x+2, y\pm1/2, -z+5/2$	H···F	6.902	−3.1	−1.1	−5.6	4.2	−5.6	−2.8
	E	$x\pm1, y\pm1, z$	F···F	8.585	1.8	−0.1	−1.1	0.1	0.8	0.4
**1,3,5F,**	A	$x, -y+3/2, z\pm1/2$	π···π	3.706	−4.9	−1.6	−21.5	17.4	−10.6	−5.3
**PVVAWA01**	B	$x\pm1/2, -y+2, z$	H···F, H···F	6.547	−6.2	−1.6	−8.4	5.9	−10.2	−5.1
*I*2/*a*	C	$x\pm1, -y+3/2, z\pm1/2$	H···F, H···F	6.844	−7.9	−1.6	−7.7	7.8	−9.5	−4.8
Z = 4,	D	$x\pm1/2, -y+1, z$	H···F, H···F	6.872	−3.2	−0.6	−6.2	3.1	−6.9	−3.5
Z' = ½	E	$x\pm1, y, z$	F···F, H···H	6.096	0.2	−0.3	−6.0	1.5	−4.6	−2.3
U = −45.8	F	$x-1/2, y\pm1/2, z-1/2$<br>$x+1/2, y\pm1/2, z+1/2$	F···F, H···H	7.529	1.2	−0.1	−2.0	0.1	−0.8	−0.4
**1,2,4,5F,**	A	$x\pm1, y, z$	π···π	4.428	−3.0	−1.5	−16.7	11.3	−9.9	−5.0
**FACJAU**<br>$P2_1/c$	B	$-x+2, y\pm1/2, -z+1/2$<br>$-x+4, y\pm1/2, -z+3/2$	F···H···F	6.892	−4.9	−1.5	−7.4	5.7	−8.1	−4.1
Z = 2	C	$x\pm1, y, z\pm1$	H···F, H···F	6.593	−3.2	−0.5	−6.3	2.6	−7.4	−3.7
Z' = ½<br>*U* = −49.1	D	$-x+3, y\pm1/2, -z+1/2$<br>$-x+3, y\pm1/2, -z+3/2$	H···F	6.051	−0.1	−0.5	−7.5	2.5	−5.6	−2.8

6. Inelastic Neutron Scattering plots

Inelastic neutron scattering plots were made using AbINS through the Mantid workbench GUI.[4] Temperatures used for simulations were taken from the experimental data log and were: **1F**- 24 K, **1,2F**- 26 K, **1,4F**- 20 K, **1,3,5F**- 30 K and **1,2,4,5F**- 20 K. AbINS applies a semi-empirical powder averaging model to the relative atomic displacements and frequencies from the phonon calculations. In this model every phonon is treated as an independent quantum harmonic oscillator.[5] Atoms are weighted by their neutron scattering cross-sections. Fundamental quantum modes up to 10 were considered, each increasing order having diminishing contribution. The specific instrument resolution function for the TOSCA instrument is built into AbINS and this was used to convolute the theoretical spectra. AbINS also removes experimentally observed overtones and accounts for the lower scattering atom types in the experimental data. Scaling of intensities was completed visually for peaks closest 1000 cm^{-1}. Data for averages of the front and back detector were used. Comparisons of experimental and simulated INS for all structures over smaller and larger spectral ranges are given below.

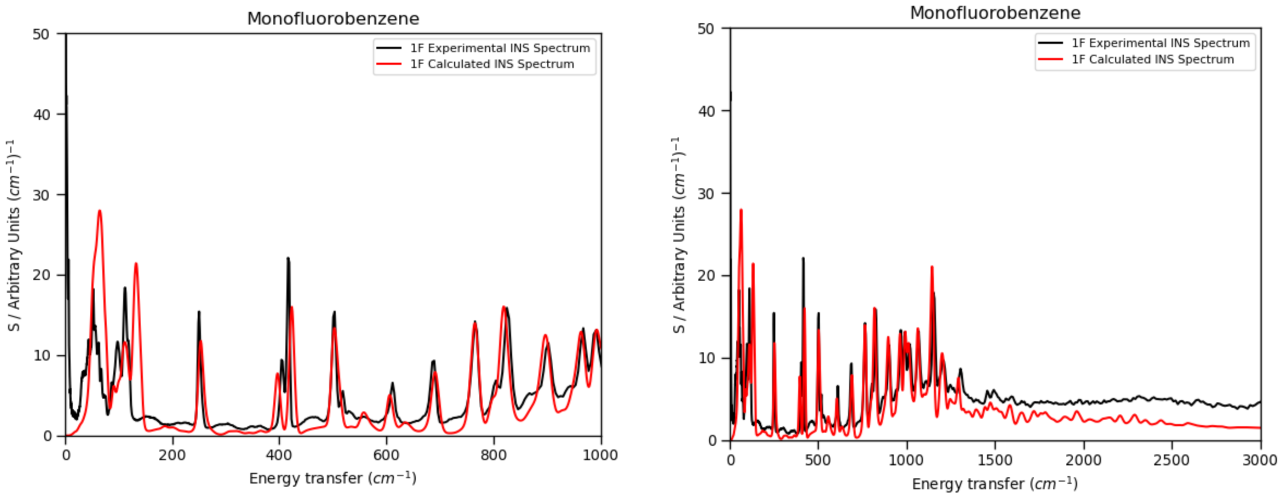

Figure S9: Comparison of experimental and calculated INS spectra for **1F** (monofluorobenzene), FACFAQ.

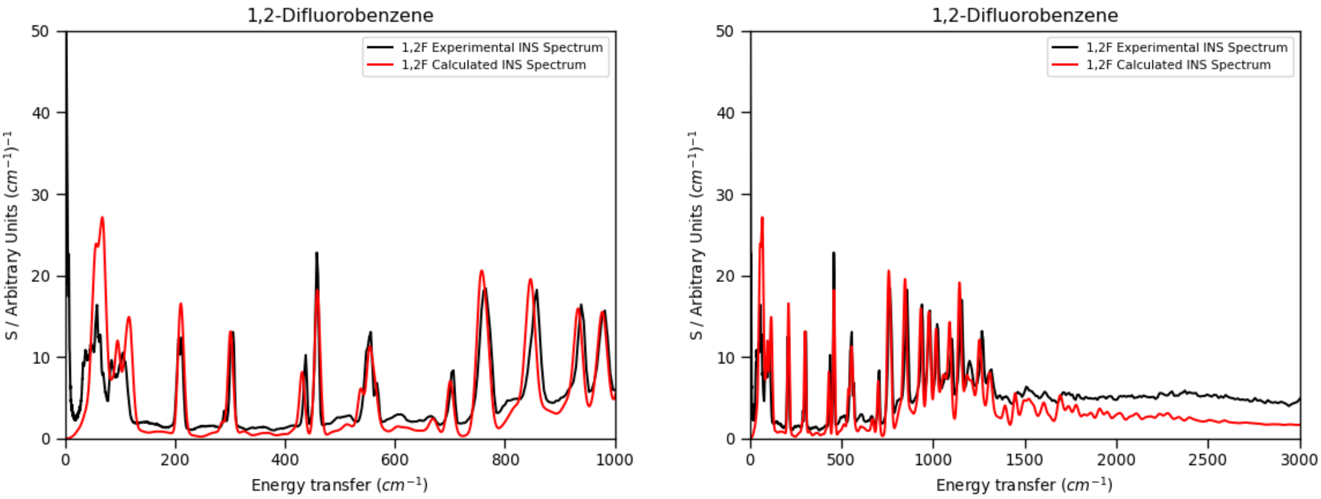

Figure S10: Comparison of experimental and calculated INS spectra for **1,2F** (1,2-difluorobenzene), FACFOE.

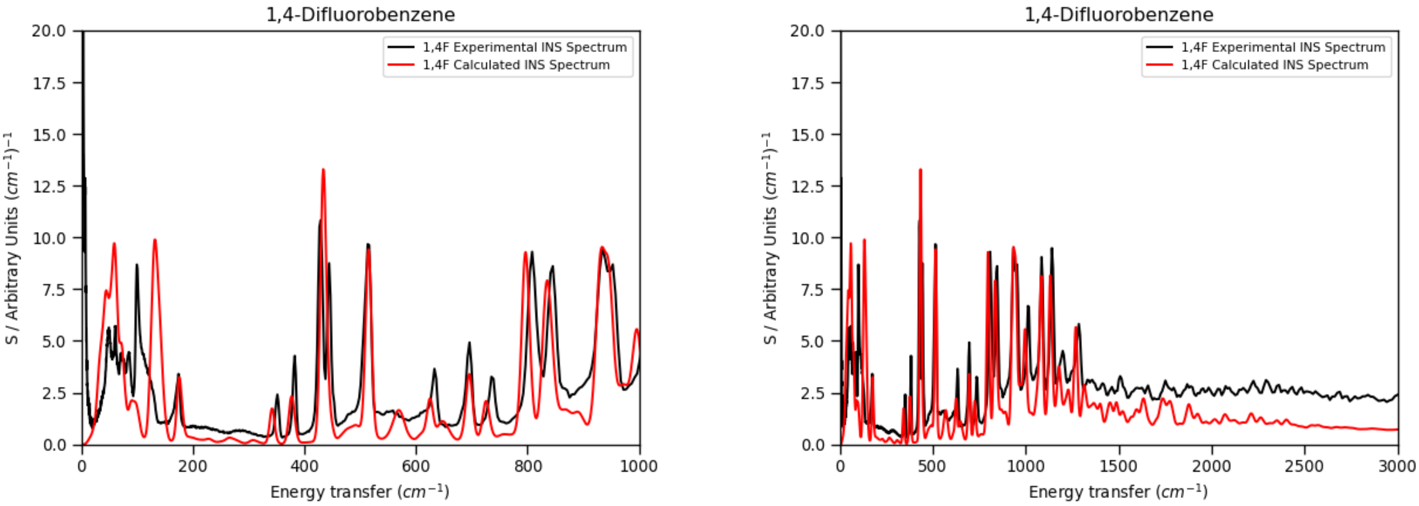

Figure S11: Comparison of experimental and calculated INS spectra for **1,4F** (1,4-difluorobenzene), FACGEV.

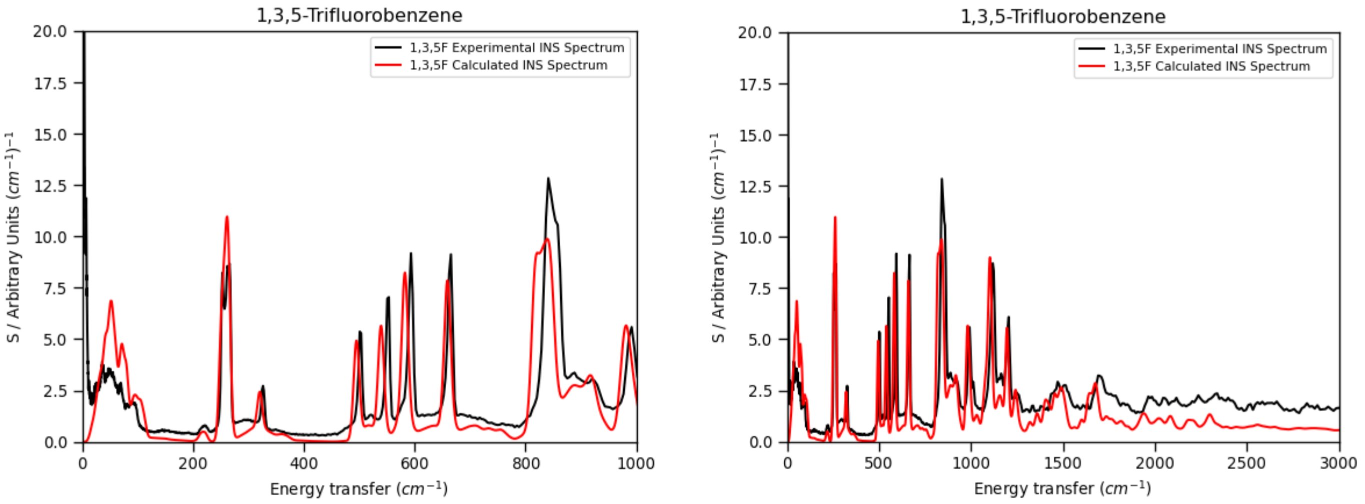

Figure S12: Comparison of experimental and calculated INS spectra for **1,3,5F** (1,3,5-trifluorobenzene), PVVAWA01.

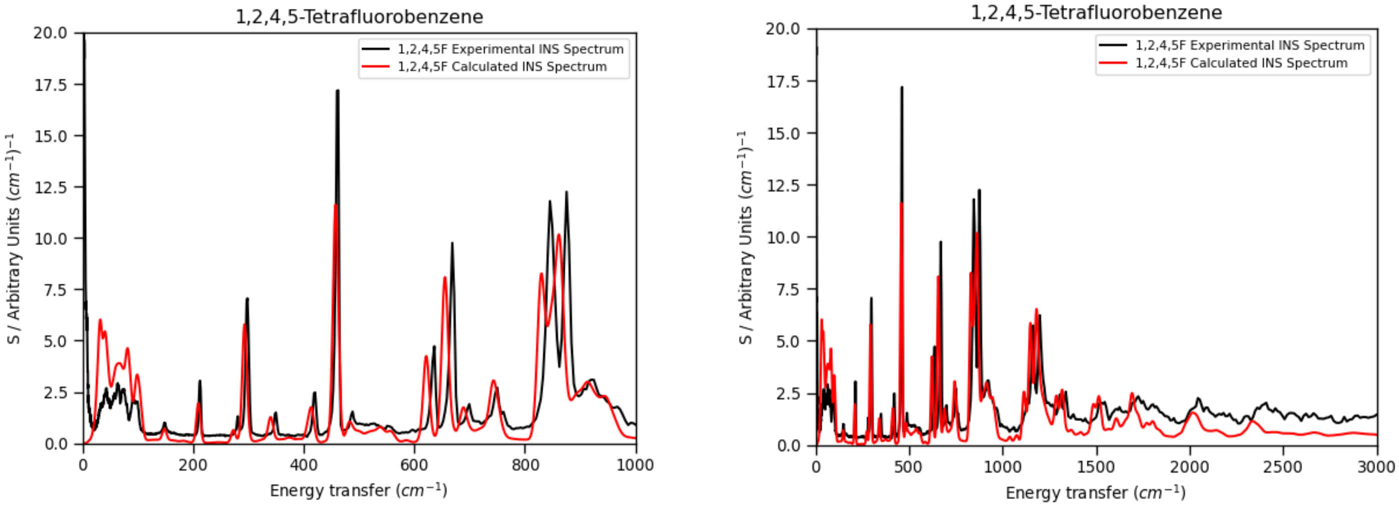

Figure S13: Comparison of experimental and calculated INS spectra for **1,2,4,5F** (1,2,4,5-tetrafluorobenzene), FACJAU.

7. Density of states plots

Phonon density of states plots for all structures over ranges of 0-1500 and 0-3250 cm^{-1} are presented below. These plots were produced through Gaussian smearing in Materials Studio with a defined smear width *c* of 0.1 THz (~3.3 cm^{-1}). Intensities of all plots are normalised such that the total integral is equal to *3N*, where *N* is the number of atoms.

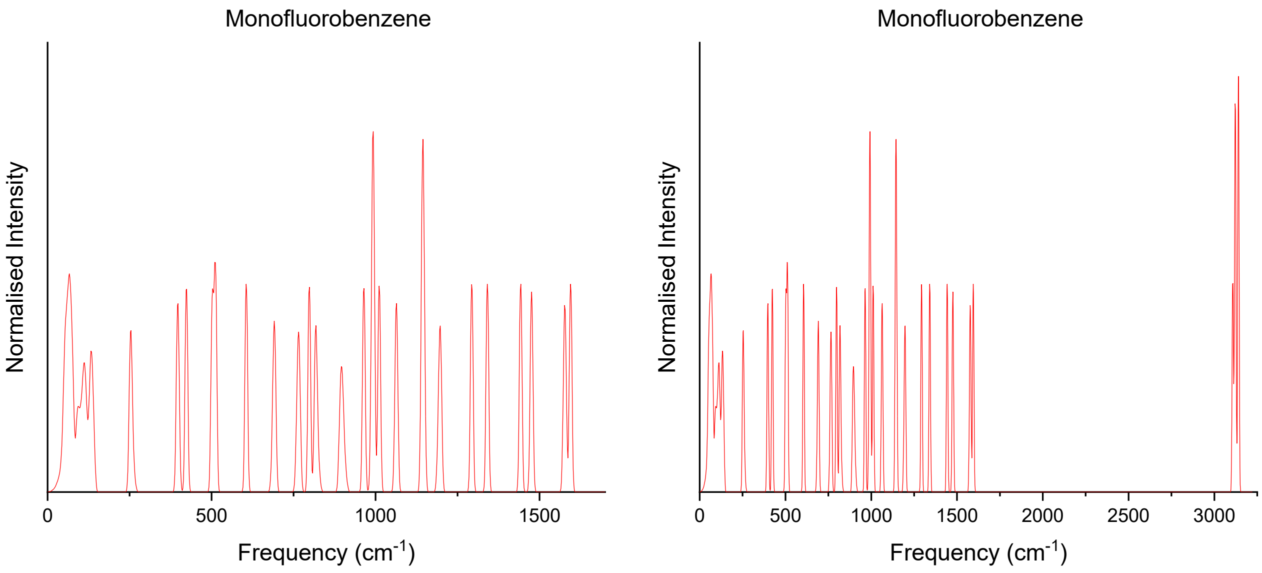

Figure S14: Density of states plots for **1F** (monofluorobenzene), FACFAQ.

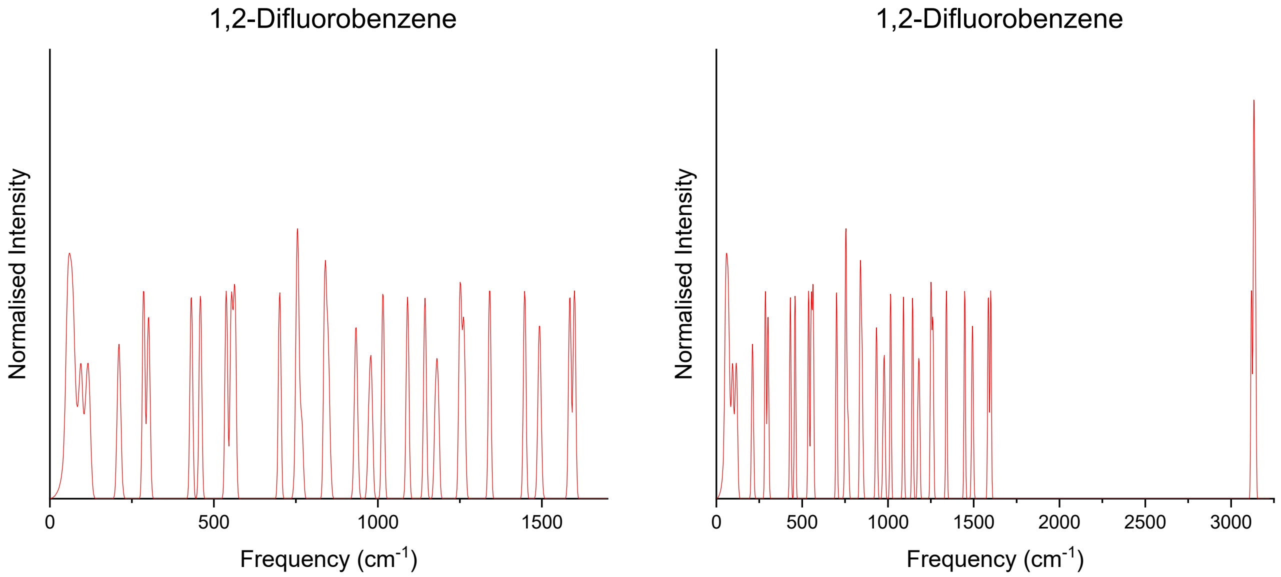

Figure S15: Density of states plots for **1,2F** (1,2-difluorobenzene), FACFOE.

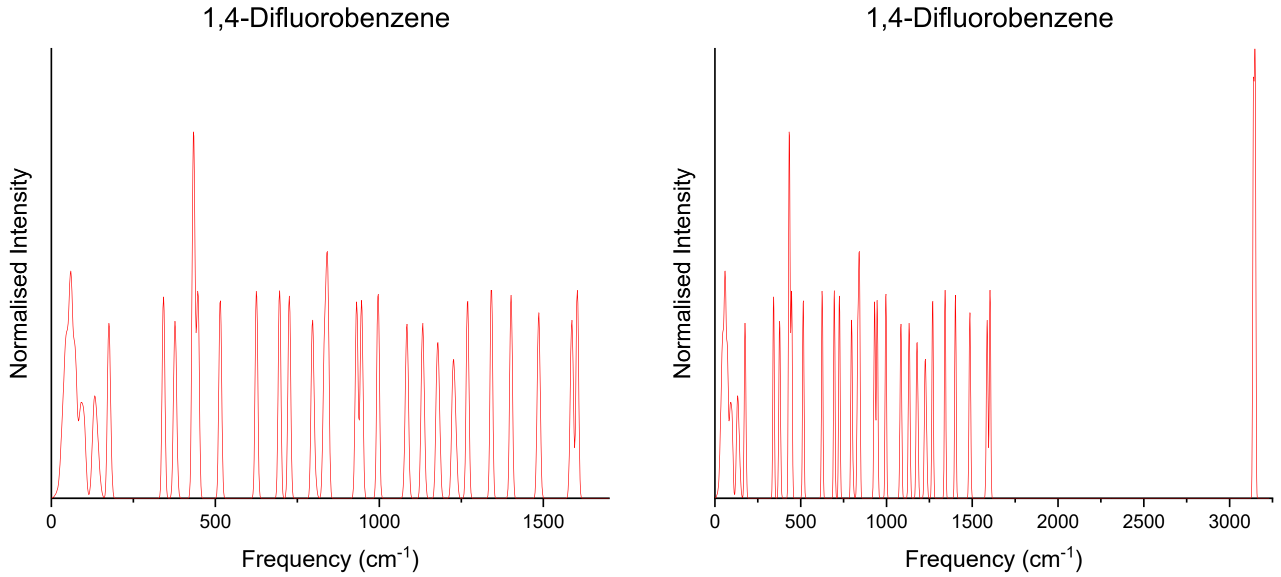

Figure S16: Density of states plots for **1,4F** (1,4-difluorobenzene), FACGEV.

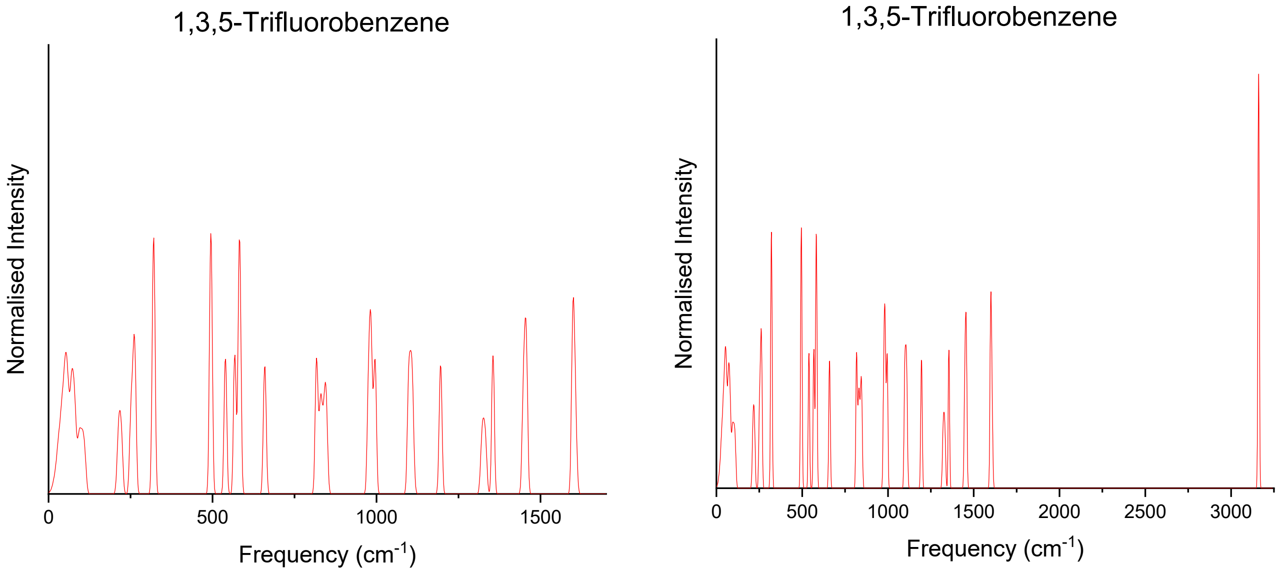

Figure S17: Density of states plots for **1,3,5F** (1,3,5-trifluorobenzene), PVVAWA01.

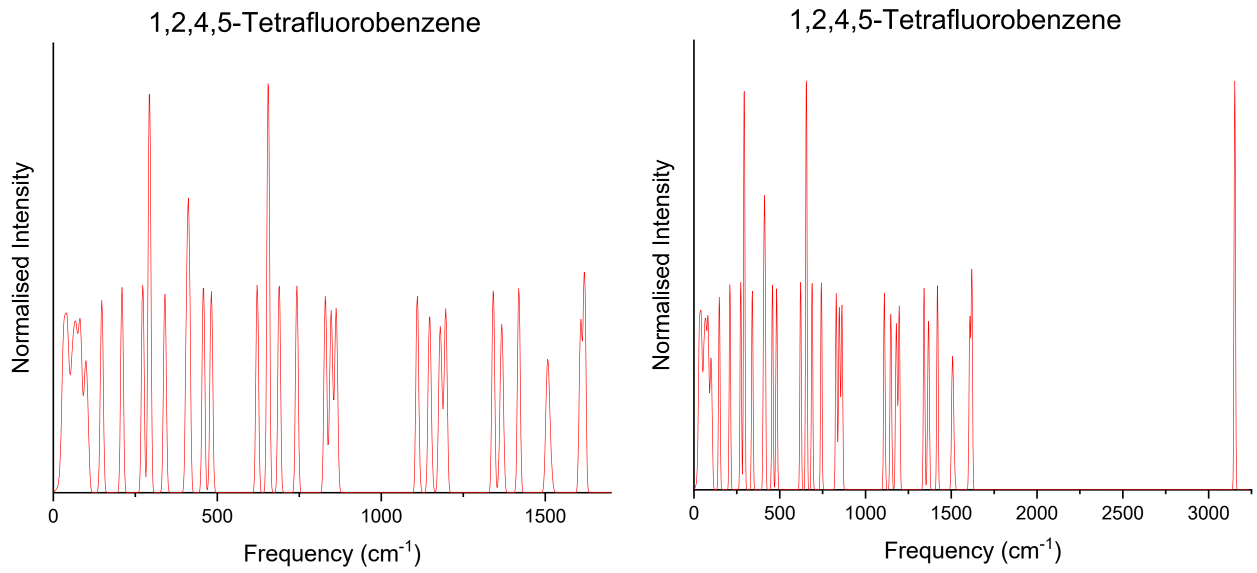

Figure S18: Density of states plots for **1,2,4,5F** (1,2,4,5-tetrafluorobenzene), FACJAU.

8. Partial density of states plots

Partial density of states plots for selected atom(s) were derived from phonons at the Γ-point.

The output of the phonon calculation contains *N*, where *N* is the number of atoms in the model, eigenvector descriptions for *x*, *y* and *z* perturbations in atom positions for each of the *3N* vibrational modes. For each eigenvector the fraction of the total motion attributed to the selected atom(s) is calculated and used to provide a total fractional contribution towards each vibrational mode. The partial density of states plots were produced via the same Gaussian smearing regime described above. The height of the resulting peaks represents the fraction of motion attributed to the selected atom type. Partial density of states plots for all structures at the Γ-point are presented below in the same ranges as the total density of states plots above.

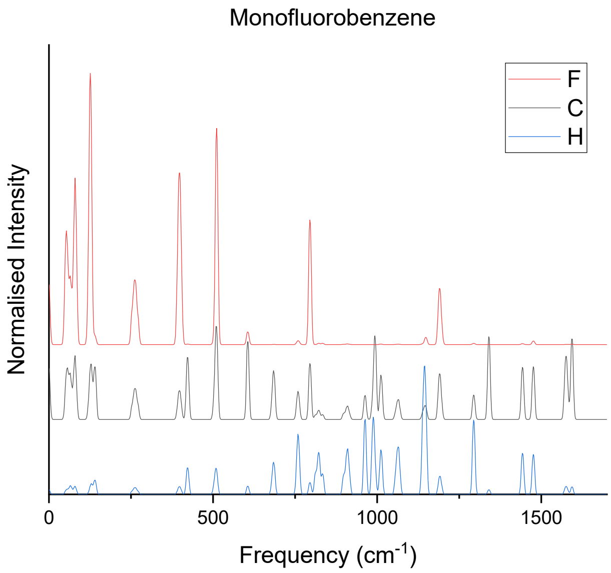

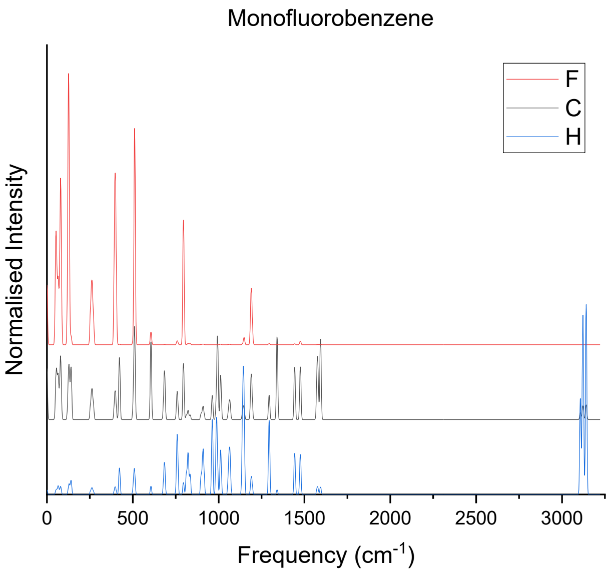

Figure S19: Partial density of states plots for **1F** (monofluorobenzene), FACFAQ.

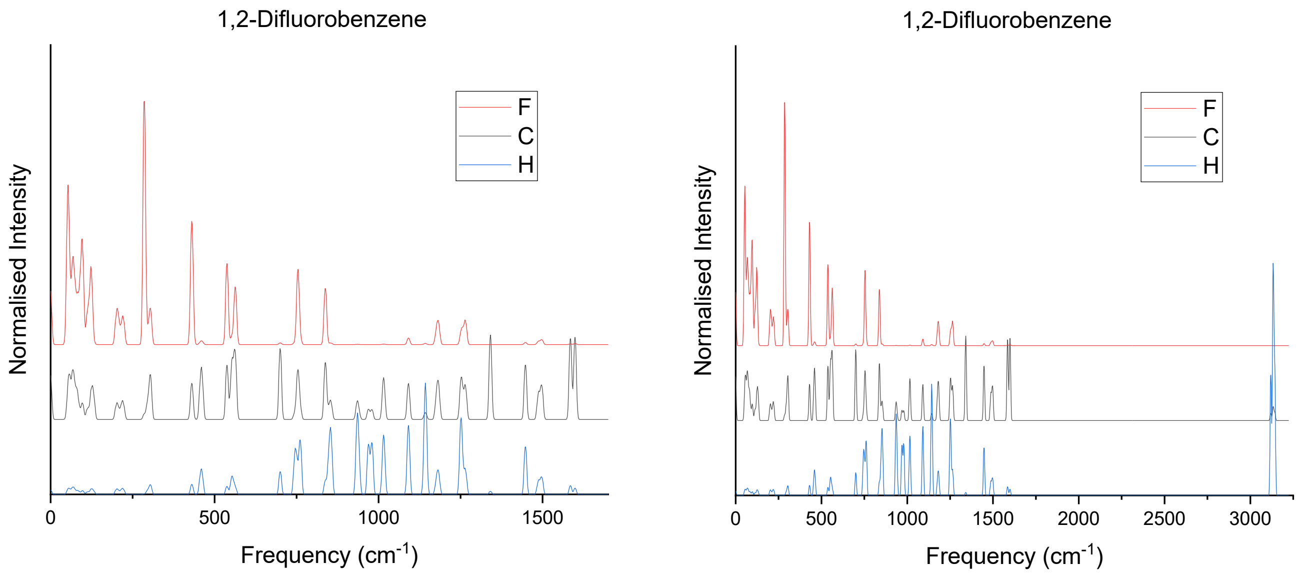

Figure S20: Partial density of states plots for **1,2F** (1,2-difluorobenzene), FACFOE.

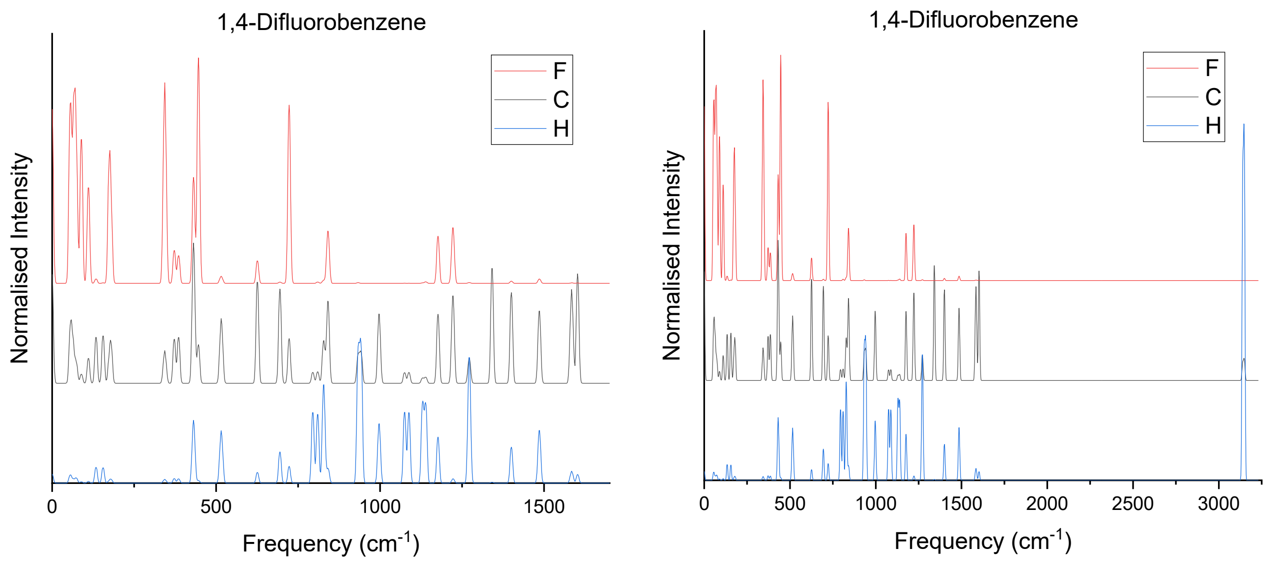

Figure S21: Partial density of states plots for **1,4F** (1,4-difluorobenzene), FACGEV.

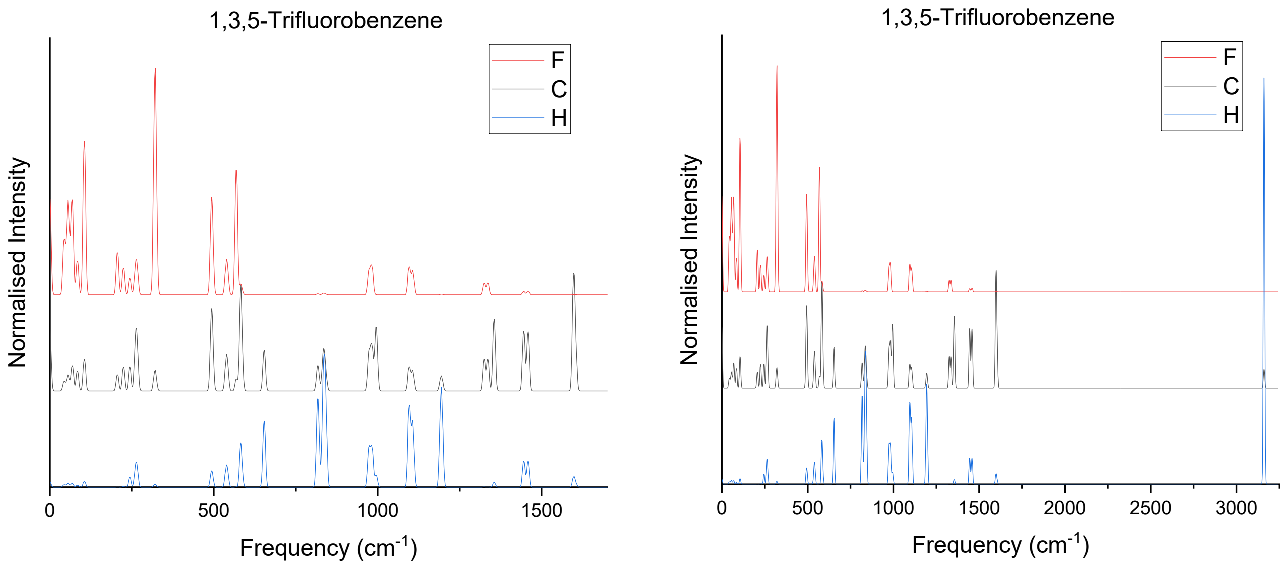

Figure S22: Partial density of states plots for **1,3,5F** (1,3,5-trifluorobenzene), PVVAWA01.

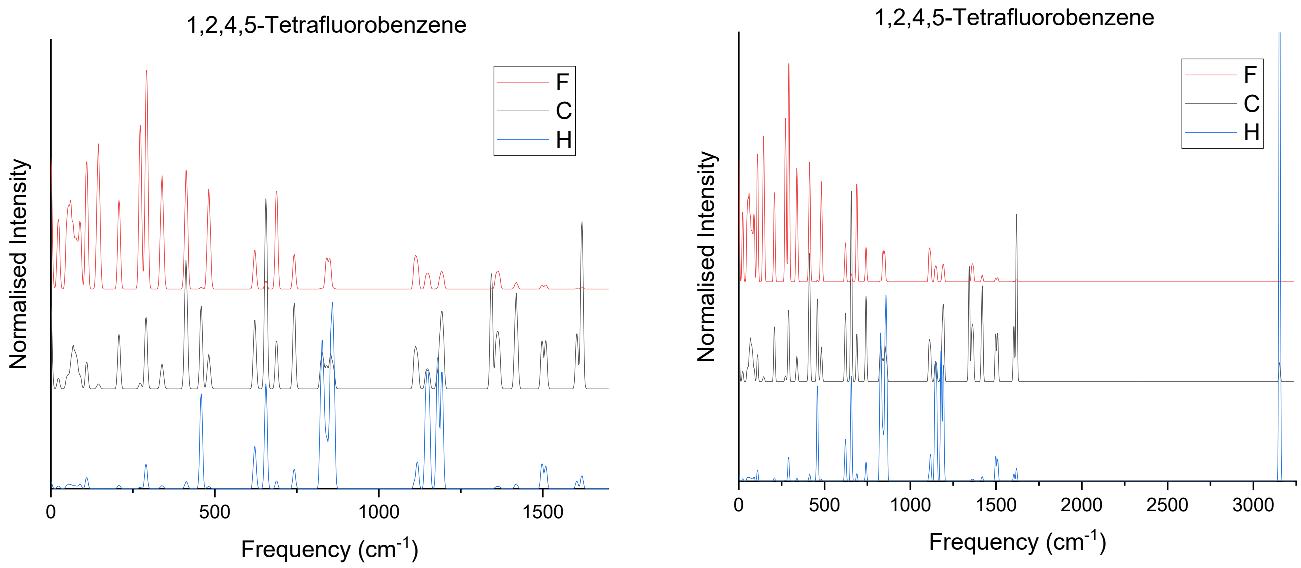

Figure S23: Partial density of states plots for **1,2,4,5F** (1,2,4,5-tetrafluorobenzene), FACJAU.

*Example: Calculation of the contributions to internal energy and entropy by occupation of energy levels at 150 K in **1,2F***

The vibrational frequencies ν at the Γ-point were obtained by periodic DFT as described in Section 2.3 of the main text. Calculation of the contribution to the entropy (S) and thermal energy (U) from each vibration at a temperature T = 150 K is accomplished using the following

equations, where *k*, *h* and *R* are Boltzmann's constant, Plank's constant and the gas constant and the value of ν in Hz is obtained from the mode wavenumber (ω in cm^{-1}) as equal to 100*c*ω, where *c* is the speed of light:

$$\theta = \frac{h\nu}{k}$$

$$U = \frac{R\theta}{\exp(\theta / T) - 1}$$

$$\frac{S}{R} = \frac{\theta / T}{\exp(\theta / T) - 1} - \ln(1 - \exp(-\theta / T))$$

The values of *U* and *TS* were calculated for each of the 141 non-acoustic modes as shown in Table S6. The magnitude of the total entropy calculated at the Γ-point (Table 2 in the main text) is obtained by summing the values in the *TS* column.

Table S6: Calculation of vibrational contributions to internal energy (*U*) and entropy (*S*) at temperature *T* = 150 K. The zero-frequency acoustic modes (1-3) have been omitted from the calculation.

	ω/ cm^{-1}	*U*/ $kJmol^{-1}$	*TS*/ $kJmol^{-1}$
4	50.387	0.970	2.166
5	52.680	0.958	2.112
6	54.537	0.949	2.069
7	55.257	0.946	2.053
8	57.727	0.934	2.000
9	62.390	0.911	1.906
10	65.167	0.898	1.853
11	68.598	0.882	1.791
12	69.426	0.878	1.777
13	72.310	0.864	1.728
14	77.293	0.841	1.649
15	80.153	0.829	1.605
16	86.037	0.803	1.522
17	92.702	0.774	1.434
18	97.617	0.753	1.374
19	99.402	0.746	1.353
20	112.082	0.695	1.215
21	120.809	0.661	1.131
22	124.301	0.648	1.099
23	125.096	0.645	1.092
24	132.490	0.618	1.029
25	200.273	0.411	0.609
26	206.649	0.395	0.580
27	216.475	0.371	0.538
28	223.340	0.355	0.511
29	284.909	0.237	0.321
30	285.158	0.237	0.320
31	285.516	0.236	0.319
32	285.785	0.236	0.319
33	295.341	0.221	0.297
34	301.758	0.211	0.282
35	305.260	0.206	0.275
36	305.625	0.206	0.274
37	429.554	0.085	0.105
38	430.007	0.085	0.105
39	431.235	0.084	0.104
40	432.977	0.083	0.102
41	456.531	0.069	0.085
42	456.764	0.069	0.085
	ω/	*U*/	*TS*/

	cm^{-1}	$kJmol^{-1}$	$kJmol^{-1}$
43	461.892	0.067	0.082
44	462.272	0.066	0.081
45	536.993	0.037	0.045
46	537.001	0.037	0.045
47	538.370	0.037	0.044
48	538.978	0.037	0.044
49	550.182	0.034	0.040
50	551.971	0.033	0.040
51	555.564	0.032	0.038
52	556.017	0.032	0.038
53	561.972	0.031	0.036
54	562.823	0.031	0.036
55	563.466	0.030	0.036
56	564.468	0.030	0.036
57	699.186	0.010	0.012
58	699.642	0.010	0.012
59	699.814	0.010	0.012
60	699.985	0.010	0.012
61	743.165	0.007	0.008
62	748.325	0.007	0.008
63	752.538	0.007	0.008
64	753.831	0.007	0.007
65	754.133	0.007	0.007
66	756.503	0.006	0.007
67	761.356	0.006	0.007
68	761.549	0.006	0.007
69	836.760	0.003	0.004
70	837.175	0.003	0.004
71	838.342	0.003	0.004
72	839.018	0.003	0.004
73	847.352	0.003	0.003
74	850.503	0.003	0.003
75	855.783	0.003	0.003
76	856.147	0.003	0.003
77	933.550	0.001	0.002
78	933.686	0.001	0.002
79	936.992	0.001	0.002
80	939.419	0.001	0.002
81	968.447	0.001	0.001
82	969.878	0.001	0.001
83	980.077	0.001	0.001
84	980.241	0.001	0.001
85	1013.351	0.001	0.001

	ω/ cm^{-1}	*U*/ kJmol^{-1}	*TS*/ kJmol^{-1}
86	1014.100	0.001	0.001
87	1016.232	0.001	0.001
88	1017.029	0.001	0.001
89	1089.389	0.000	0.000
90	1089.934	0.000	0.000
91	1091.601	0.000	0.000
92	1092.953	0.000	0.000
93	1140.712	0.000	0.000
94	1141.424	0.000	0.000
95	1143.964	0.000	0.000
96	1145.049	0.000	0.000
97	1174.767	0.000	0.000
98	1179.551	0.000	0.000
99	1181.671	0.000	0.000
100	1184.284	0.000	0.000
101	1249.477	0.000	0.000
102	1250.856	0.000	0.000
103	1251.636	0.000	0.000
104	1251.882	0.000	0.000
105	1255.445	0.000	0.000
106	1260.825	0.000	0.000
107	1265.812	0.000	0.000
108	1266.429	0.000	0.000
109	1340.613	0.000	0.000
110	1340.917	0.000	0.000
111	1341.586	0.000	0.000
112	1341.688	0.000	0.000
113	1447.824	0.000	0.000
114	1447.854	0.000	0.000
115	1447.893	0.000	0.000
116	1448.364	0.000	0.000
117	1484.981	0.000	0.000
118	1490.357	0.000	0.000
119	1496.781	0.000	0.000
120	1498.169	0.000	0.000
121	1584.175	0.000	0.000
122	1585.079	0.000	0.000
123	1585.394	0.000	0.000
124	1585.517	0.000	0.000
125	1598.034	0.000	0.000
126	1598.538	0.000	0.000
127	1599.391	0.000	0.000
128	1600.502	0.000	0.000

	ω/ cm^{-1}	*U*/ kJmol^{-1}	*TS*/ kJmol^{-1}
129	3118.791	0.000	0.000
130	3118.986	0.000	0.000
131	3119.141	0.000	0.000
132	3119.381	0.000	0.000
133	3131.218	0.000	0.000
134	3131.230	0.000	0.000
135	3131.704	0.000	0.000
136	3131.721	0.000	0.000
137	3134.187	0.000	0.000
138	3134.643	0.000	0.000
139	3134.727	0.000	0.000
140	3135.236	0.000	0.000
141	3141.774	0.000	0.000
142	3142.085	0.000	0.000
143	3142.512	0.000	0.000
144	3143.433	0.000	0.000
Total/cell		21.669	39.985
Total/mol		5.417	9.996

9. Justification for the neglect of *U* and zero-point energy

The equations for *U* and *TS* given in Section 8 can be plotted as a function of ω for *T* = 150 K to allow the influence of these quantities on the free energy to be compared (Fig. S24). The plot shows that *TS* is much more sensitive to the values of the frequencies of low-energy vibrational modes than is *U*. Small differences in external mode vibrational frequencies make little difference to the contribution of mode to *U*, and alternative packing arrangements will have similar values of *U*. This reflects the observation made by Nyman and Day[6] that the difference in heat capacity between polymorphs are very small.

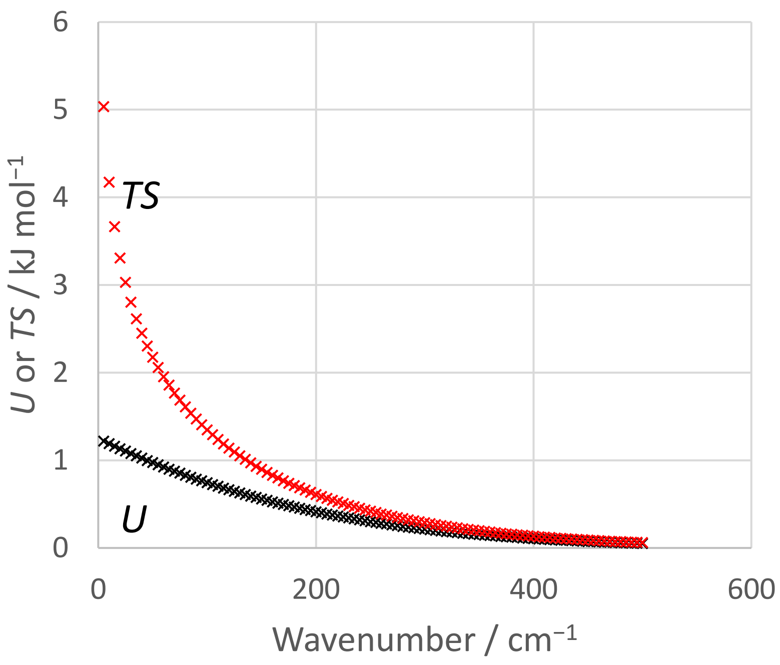

Figure S24. Comparison of the contributions of low-energy vibrational modes to entropy and internal energy with *T* = 150 K.

In the harmonic approximation the zero-point energy is given by ½Σ*hv* and is dominated by the highest energy internal vibrational modes. These modes can vary between polymorphs, but usually not by much and differences are a minor contributor to the free energy differences between polymorphs,[6] though they can assume significance in the cases where strong intermolecular interactions such as H-bonds are present as these can perturb internal bonds more substantially than the weak interactions that are considered in the present work.[7]

References

(1) Groom, C. R.; Bruno, I. J.; Lightfoot, M. P.; Ward, S. C. The Cambridge Structural Database. *Acta Crystallogr., Sect. B: Struct. Sci., Cryst. Eng. Mater.* **2016**, *72* (2), 171-179.

(2) Jeziorski, B.; Moszynski, R.; Szalewicz, K. Perturbation Theory Approach to Intermolecular Potential Energy Surfaces of van der Waals Complexes. *Chem. Rev.* **1994**, *94* (7), 1887-1930. Smith, D. G. A.; Burns, L. A.; Simmonett, A. C.; Parrish, R. M.; Schieber, M. C.; Galvelis, R.; Kraus, P.; Kruse, H.; Di

Remigio, R.; Alenaizan, A.; et al. PSI4 1.4: Open-Source Software for High-Throughput Quantum Chemistry. *J. Chem. Phys.* **2020**, *152* (18), 184108.
(3) Parker, T. M.; Burns, L. A.; Parrish, R. M.; Ryno, A. G.; Sherrill, C. D. Levels of Symmetry Adapted Perturbation Theory (SAPT). I. Efficiency and Performance for Interaction Energies. *J. Chem. Phys.* **2014**, *140* (9), 094106.
(4) Dymkowski, K.; Parker, S. F.; Fernandez-Alonso, F.; Mukhopadhyay, S. AbINS: The Modern Software for INS Interpretation. *Physica B* **2018**, *551*, 443-448.
(5) Armstrong, J.; O'Malley, A. J.; Ryder, M. R.; Butler, K. T. Understanding Dynamic Properties of Materials Using Neutron Spectroscopy and Atomistic Simulation. *J. Phys. Commun.* **2020**, *4* (7), 072001.
(6) Nyman, J.; Day, G. M. Static and Lattice Vibrational Energy Differences between Polymorphs. *CrystEngComm* **2015**, *17* (28), 5154-5165.
(7) Rivera, S. A.; Allis, D. G.; Hudson, B. S. Importance of Vibrational Zero-Point Energy Contribution to the Relative Polymorph Energies of Hydrogen-Bonded Species. *Cryst. Growth Des.* **2008**, *8* (11), 3905-3907.
